# Development of Extracellular Vesicle Therapeutics: Challenges, Considerations, and Opportunities

**DOI:** 10.3389/fcell.2021.734720

**Published:** 2021-09-20

**Authors:** Bethany Claridge, Jonathan Lozano, Qi Hui Poh, David W. Greening

**Affiliations:** ^1^Department of Biochemistry and Genetics, La Trobe Institute for Molecular Science (LIMS), La Trobe University, Melbourne, VIC, Australia; ^2^Baker Heart and Diabetes Institute, Melbourne, VIC, Australia; ^3^Department of Physiology, Anatomy and Microbiology, La Trobe University, Melbourne, VIC, Australia; ^4^Central Clinical School, Monash University, Melbourne, VIC, Australia; ^5^Baker Department of Cardiometabolic Health, University of Melbourne, Melbourne, VIC, Australia

**Keywords:** extracellular vesicle therapeutics, drug and vector delivery, exosome-based therapeutics, nanomedicine, nanovesicles/microparticles, EV hybrids and mimetics, bioengineering, clincal trials and utility

## Abstract

Extracellular vesicles (EVs) hold great promise as therapeutic modalities due to their endogenous characteristics, however, further bioengineering refinement is required to address clinical and commercial limitations. Clinical applications of EV-based therapeutics are being trialed in immunomodulation, tissue regeneration and recovery, and as delivery vectors for combination therapies. Native/biological EVs possess diverse endogenous properties that offer stability and facilitate crossing of biological barriers for delivery of molecular cargo to cells, acting as a form of intercellular communication to regulate function and phenotype. Moreover, EVs are important components of paracrine signaling in stem/progenitor cell-based therapies, are employed as standalone therapies, and can be used as a drug delivery system. Despite remarkable utility of native/biological EVs, they can be improved using bio/engineering approaches to further therapeutic potential. EVs can be engineered to harbor specific pharmaceutical content, enhance their stability, and modify surface epitopes for improved tropism and targeting to cells and tissues *in vivo*. Limitations currently challenging the full realization of their therapeutic utility include scalability and standardization of generation, molecular characterization for design and regulation, therapeutic potency assessment, and targeted delivery. The fields’ utilization of advanced technologies (imaging, quantitative analyses, multi-omics, labeling/live-cell reporters), and utility of biocompatible natural sources for producing EVs (plants, bacteria, milk) will play an important role in overcoming these limitations. Advancements in EV engineering methodologies and design will facilitate the development of EV-based therapeutics, revolutionizing the current pharmaceutical landscape.

## Introduction

Extracellular vesicles (EVs) are nanosized, membranous cell-derived particles with important roles in exchanging molecular information between cells; they have been shown to contain and transfer proteins and nucleic acids (DNA, mRNA, miRNA) to recipient cells, modulating their functional activity through transcriptional and translational regulation (Luga et al., 2012; Xu et al., 2016; Thery et al., 2018; Flaherty et al., 2019; Kalluri and LeBleu, 2020); transfer of organelles has also been highlighted by EVs, including mitochondria (Spees et al., 2006), to regulate inflammatory response (Zhang Y. et al., 2021). EVs can be classified by their biogenesis and biophysical/biochemical characteristics. The subtypes include intracellular formed exosomes (50–200 nm) secreted after fusion of multivesicular bodies with the cell surface, microvesicles (100–1,000 nm) formed by outward budding of the plasma membrane, shed midbody remnants released during cytokinesis (200–600 nm), and apoptotic bodies (100–5,000 nm) released during apoptosis (Al-Nedawi et al., 2008; Willms et al., 2016; Xu et al., 2016; van Niel et al., 2018; Jeppesen et al., 2019; Martinez-Greene et al., 2021; Rai et al., 2021). The International Society for EVs (ISEV) recommends the use of “EV” as a broad classifier term for these types of vesicles, due to the difficulty in assigning an EV to a particular biogenesis pathway, and instead recommends classifying EVs by their physical attributes (size, density), their differing biochemical composition, and surface charge (Thery et al., 2018). The nature and relative abundance of EV cargo is selectively determined during EV biogenesis (Palmulli and van Niel, 2018; van Niel et al., 2018; Clancy et al., 2021), and varies according to EV subtype and state/type of the producing cell (Kowal et al., 2016; Xu et al., 2016; Zabeo et al., 2017; Greening and Simpson, 2018; Martin-Jaular et al., 2021). Importantly, EVs, comprising of a lipid membrane and aqueous lumen (Cvjetkovic et al., 2016; Skotland et al., 2019), provide a pathway for the transfer of hydrophobic and hydrophilic components allowing for complex intercellular signaling (Luga et al., 2012; Cossetti et al., 2014; de Couto et al., 2017; Kamerkar et al., 2017; Nabet et al., 2017; Wang and Lu, 2017; Flaherty et al., 2019; Han et al., 2019).

Due to their nanoscale size, stability, biocompatibility, and propensity for cellular uptake, EVs have been recognized as viable vehicles for therapeutic application. Recent studies have highlighted the therapeutic potential of EVs, investigated in clinical trials (phase I/II) for their regenerative capacity (NCT04223622) (Niada et al., 2019), vaccine potential (Gehrmann et al., 2014; Narita et al., 2015; Besse et al., 2016; Coakley et al., 2017; Shehata et al., 2019; Nikfarjam et al., 2020; Andrews et al., 2021), immunotherapeutic activity (Narita et al., 2015; Besse et al., 2016; Chen G. et al., 2018), and application as delivery vectors (NCT01294072). Indeed, pre-clinical and therapeutic applications of EVs across a wide range of pathologies for tissue repair and regeneration are well underway (Table 1). Critically, stem cell-derived EVs can ameliorate the effects of various diseases in the liver (fibrosis, hepatitis, inflammation), brain (stroke), heart (myocardial infarction, contractility), kidney (renal ischemia, stenosis), and immune system [reviewed in Wiklander et al. (2019) and Yin et al. (2019)]. EVs have been shown in various mechanisms to regulate the immune system, enhancing or inhibiting the immune response depending on their parental cell source and of the immune context of the application site (Zhou et al., 2020), demonstrating a potential use in immunotherapy. Indeed, EVs exert specific and potent therapeutic effects on recipient cells because of complex bioactive properties and are an effective and efficient system of cell-cell communication, surmounting biological barriers.

**TABLE 1:** Clinical and preclinical applications of extracellular vesicles.

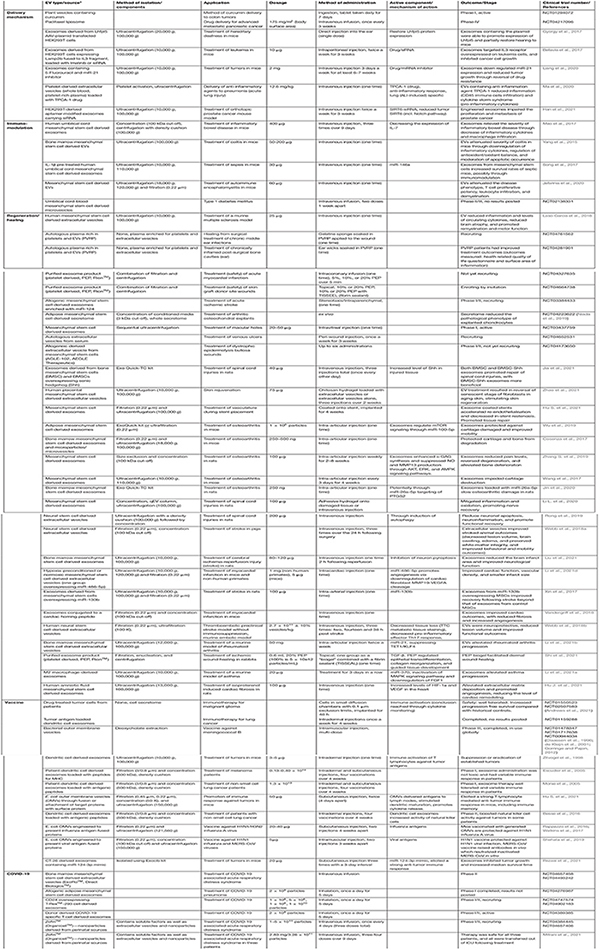

*Nomenclature presented is that used in study/trial.

The diverse beneficial effects of seemingly identical entities [i.e., mesenchymal stem cell (MSC)-EVs, bone marrow-derived stem cell (BMDSC)-EVs (Kordelas et al., 2014; de Couto et al., 2017; Xue et al., 2018; Han et al., 2019; Lukomska et al., 2019; Williams A. M. et al., 2019)] suggests a complex repertoire of active cargo (Toh et al., 2018) working synergistically, as opposed to a single molecular component. As such, a global view of EV-based therapeutic action is needed. The biological cargo harbored by EVs, including proteins (Al-Nedawi et al., 2008; Yim et al., 2016; Yuan et al., 2017; Zhang G. et al., 2017; Roefs et al., 2020), nucleic acids (Ratajczak et al., 2006; Liang et al., 2016; Xiao G. Y. et al., 2016; Song et al., 2017; Gu et al., 2018; Shi et al., 2018; Gu Y. Y. et al., 2019; Basalova et al., 2020), and lipids (Lindemann, 1989; Fadok et al., 2000; Gurnani et al., 2004; Yuyama et al., 2014) [reviewed in Greening et al. (2017), Skotland et al. (2019), O’Brien et al. (2020)] (Table 2), greatly influence their clinical potential. The protein and lipid expression of EVs yield insights into their surface receptor mediated interactions with, and effects on recipient cells, including their fusion and uptake (Christianson et al., 2013; Purushothaman et al., 2016; Berenguer et al., 2018), while their genetic landscape sheds light on the EVs’ reprogramming potential through regulation of protein expression (Ratajczak et al., 2006; Skog et al., 2008; Abels et al., 2019). Findings from such studies have inspired strategies for EV-based therapeutic development, such as the modification of their contents to perform a specified function for a specific disease phenotype (Table 3) (Al-Nedawi et al., 2008; Hall et al., 2016; Yim et al., 2016; Greening et al., 2017; Yuan et al., 2017; Zhang G. et al., 2017; Chen R. et al., 2019; Shi et al., 2019; Skotland et al., 2019; O’Brien et al., 2020; Roefs et al., 2020). Comprehensive deciphering of EV biochemical and biophysical heterogeneity (Jeppesen et al., 2019), variable composition (Chen et al., 2016; Kowal et al., 2016; Greening et al., 2017; Jeppesen et al., 2019; Martin-Jaular et al., 2021), pharmacokinetic behavior (Gupta et al., 2020), and functional diversity needs to be addressed in order to harness their potential as next generation therapeutics.

**TABLE 2:** Evidence supporting the role of extracellular vesicle cargo in intercellular communication.

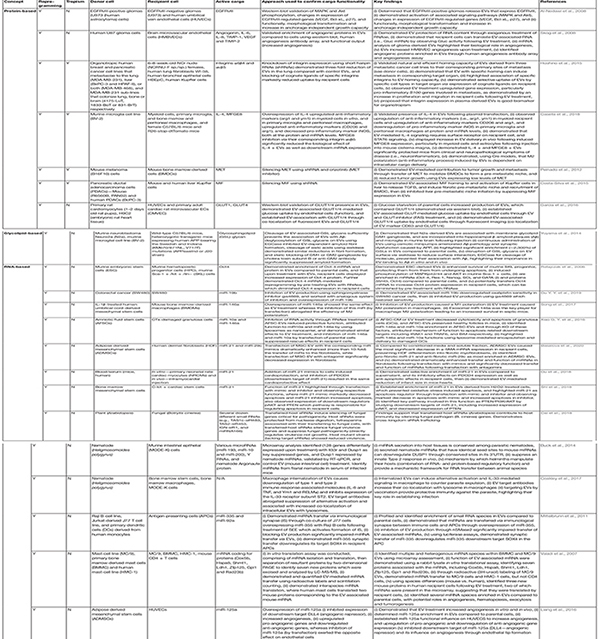

**TABLE 3:** Approaches in engineering extracellular vesicles.

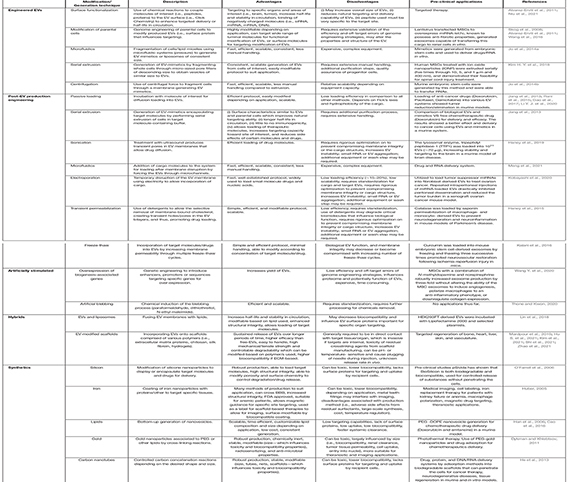

Advances and applied technologies which can be used to reproducibly monitor form and function of EVs at molecular and structural levels will be instrumental in future development of EV research knowledge, and therapeutic design and application. Bioinspired engineered EVs/nanovesicles have emerged as an alternative to native EVs to address issues in production, purity, scalability, and economic viability, while maintaining key properties required for *in vivo* trafficking, biological function, and therapeutic efficiency. Recent advances in bioengineering have allowed the refinement of cargo loading, targeting capacity, and pharmacokinetic properties of EV-based therapeutics, both native and mimetic.

Here, we focus on recent advances in clinical utility of EVs, understanding the molecular complexity of bioactive cargo, avenues for bioengineering, and monitoring the form and function of EVs intended for clinical use. For further discussion on topics not covered in extensive detail here, we direct readers to the following recent reviews and position papers (Lener et al., 2015; Reiner et al., 2017; Colao et al., 2018; Pinheiro et al., 2018; Sluijter et al., 2018; Russell et al., 2019; Whitford and Guterstam, 2019; Wiklander et al., 2019; Gandham et al., 2020; Nelson et al., 2020; Pirisinu et al., 2020; Yin et al., 2020; Grangier et al., 2021; Herrmann et al., 2021; Johnson et al., 2021; Ozturk et al., 2021; Sahoo et al., 2021; Suharta et al., 2021).

## Current Developments in EV-Based Therapeutics

Recent years have seen significant development and translation of EV-associated therapeutics, progressing to pre-clinical and clinical studies (Table 1). Further, the capacity of EVs to transfer biological and pharmaceutical molecules to specific tissues and cell types has raised considerable interest in their development as biocompatible drug delivery systems [reviewed in Sluijter et al. (2018); Pirisinu et al. (2020); Herrmann et al. (2021); and Rankin-Turner et al. (2021)]. At present, https://www.clinicaltrials.gov lists 224 studies which include “exosome,” 84 with “extracellular vesicle,” 9 with “nanocarrier,” 4 with “engineered exosome,” and 2,101 with “liposome.” While a portion of these are diagnostic/biomarker studies, most are clinical trials based on pre-clinical therapeutic success in wound healing (NCT04761562, NCT04281901, and NCT04664738) (Jia et al., 2021; Zhao et al., 2021), heart disease (NCT04327635) (Aday et al., 2021; Hu S. et al., 2021), COVID-19 (NCT04657458, NCT04493242, NCT04276987, NCT04747574, NCT04389385, and NCT04969172) (Mitrani et al., 2021), infectious disease (NCT01478347, NCT01717638, and NCT04350138), diabetes (NCT02138331), stroke (NCT03384433), arthritis (NCT04223622), and drug delivery (NCT01294072, NCT02889822, and NCT04217096). While classified as EV therapies, such studies are more often comprised of a variety of secreted components (i.e., secretome containing soluble factors and EVs) than purified EVs (Table 1). Various terminology is used in the field (Thery et al., 2018), including “extracellular vesicles,” “exosomes,” “secretomes,” “nanoparticles,” or components “enriched in extracellular vesicles.” Regardless of terminology or composition, these therapies utilize the functional capacity EVs/secreted components have to mediate a recipient-cell response through the delivery of cargo including siRNAs (Shtam et al., 2013), miRNAs (Li L. et al., 2019), proteins (Garaeva et al., 2021), small molecule drugs (Tian et al., 2014), and molecular toolkits (Ye et al., 2020; Luo et al., 2021; Yao X. et al., 2021).

### Immunomodulation

Extracellular vesicles hold the potential for potent immunomodulation, both in eliciting and suppressing immune response (Zhou et al., 2020). EVs share structural similarities to viruses and recent findings demonstrate that viruses exploit mechanisms associated with EV uptake and release (Feng et al., 2013; Altan-Bonnet, 2016; van Dongen et al., 2016; Urbanelli et al., 2019). Previously, the ground-breaking application of EVs in anti-tumor immunotherapy (Zitvogel et al., 1998) led to two clinical trials where EVs activated patient immune response against tumor antigens (Escudier et al., 2005; Morse et al., 2005). Since then, refinements in EV production and modification have led to successful reduction in tumor size in various pre-clinical models (Lee et al., 2012; Mahaweni et al., 2013; Rao et al., 2016; Cheng et al., 2021) and additional clinical trials exploiting their immunomodulatory capabilities to target various cancer types (NCT01550523, NCT01159288, and NCT02507583) (Besse et al., 2016; Andrews et al., 2021). The use of EVs as antigen vehicles is an approach still under development (Cheng et al., 2021; Hu S. et al., 2021), but does represent the most successful translated application. The FDA approved Bexsero bacterial outer membrane vesicle (OMV)-containing meningococcal vaccine is administered to protect against meningococcal group B (Gorringe and Pajon, 2012). This vaccine exploits the complexity of bacterial OMVs to boost immunogenicity of multi-component formulas over those without OMVs (Gorringe and Pajon, 2012). Anti-viral EV-based vaccines are also in development (Sabanovic et al., 2021), with pre-clinical success against COVID-19 (Polak et al., 2020), influenza H1N1 (Rappazzo et al., 2016; Watkins et al., 2017; Shehata et al., 2019), influenza H3N2 (Watkins et al., 2017), and MERS-CoV (Shehata et al., 2019). In addition to immune-priming, EV therapeutics hold the potential for deactivation and suppression of over-active immune responses. Indeed, although results have yet to be released, a clinical trial is underway examining the therapeutic effect of MSC-derived EVs in the autoimmune disease Type I diabetes (NCT02138331). Pre-clinical success in the treatment of sepsis (Song et al., 2017), inflammatory bowel disease (Yang et al., 2015; Mao et al., 2017), and multiple sclerosis (Casella et al., 2018; Laso-Garcia et al., 2018) suggests EV-mediated treatment of inflammatory and autoimmune disorders (Coakley et al., 2017; Sharma et al., 2017; Fujita et al., 2018; Xu H. et al., 2019; Goodman and Davies, 2020; Kahmini and Shahgaldi, 2020; Xu et al., 2020; Horst et al., 2021) may soon expand further into patient trials.

### Regeneration and Recovery

Extracellular vesicle therapeutics are also being explored to facilitate/promote recovery and regeneration following injury, surgery, and tissue damage, such as that arising from viral infections [i.e., pulmonary damage in COVID-19 (Borczuk et al., 2020)]. There are currently 13 clinical trials treating COVID-19 with “exosomes” or “extracellular vesicles” (NCT04902183, NCT04798716, NCT04602442, NCT04747574, NCT04491240, NCT04389385, NCT04276987, NCT04657406, NCT04384445, NCT04623671, NCT04493242, NCT04657458, and NCT04969172). Although no large studies have released results, a case report of three critically ill patients administered with amniotic fluid-derived nanoparticles (likely containing EVs and other small extracellular particles) revealed the therapy had no adverse effects and the patients’ status improved (Mitrani et al., 2021). There are now clinical trials further evaluating the safety and efficacy of this treatment for COVID-19 (NCT04384445, NCT04657406). Another major application of EV therapies is in accelerated and improved healing and regeneration of damaged tissue (Dalirfardouei et al., 2021; Hu S. et al., 2021; Jia et al., 2021; Lou et al., 2021; Saludas et al., 2021; Zhao et al., 2021). There are promising results in the utilization of EVs to repair arthritic joints (NCT04223622) (Cosenza et al., 2017; Wang et al., 2017; Wu et al., 2019; Zhang S. et al., 2019; Jin et al., 2020) and spinal-cord injuries (Rong et al., 2019; Li L. et al., 2020; Jia et al., 2021), with tissue restoration in EV treatments exceeding that of untreated animals/tissue. As these are recent developments, mechanism of action and clinical translation are still underway. However, there are many clinical trials addressing the regenerative capacity of EVs in surgical and dermal wound healing and tissue regeneration (NCT04652531, NCT04173650, NCT04664738, NCT04761562, and NCT04281901). The regenerative capacity of EVs is also relevant in rescuing and restoring function in damaged organs, including brain (Galieva et al., 2019; Beard et al., 2020) and heart (Kennedy et al., 2020). Animal models of stroke treated with EVs show improved outcomes (Doeppner et al., 2015; Otero-Ortega et al., 2017; Tian et al., 2018; Webb et al., 2018a; Spellicy et al., 2020; Liu et al., 2021), in particular those derived from MSCs overexpressing miR-133b (Xin et al., 2017). Indeed, overexpression of miRNAs in EVs represents a promising avenue, with a clinical trial administering EVs enriched with miR-124 for stroke recovery underway (NCT03384433). Cardiac damage following myocardial infarction is debilitating and previously un-preventable, however, there has been significant preclinical success using EVs in reducing infarct size and restoring cardiac function in various *in vivo* models (Barile et al., 2014; Bian et al., 2014; Chen C. W. et al., 2018; Vandergriff et al., 2018; Hu S. et al., 2021; Saludas et al., 2021; Zhu et al., 2021b). There is now a clinical trial treating patients with an EV product following acute myocardial infarction (NCT04327635). While we wait for results from these trials to be released, the applications and modulation of EV therapies will continue to be developed for the next phase.

### Combination Therapies

The therapeutic and clinical potential of EVs is further elevated through their biocompatibility and capacity to be surface modified and coupled with other remedial approaches. As discussed, protein or nucleic acid landscape can be modulated in parental cells to generate vaccines (Rappazzo et al., 2016; Watkins et al., 2017; Shehata et al., 2019; Polak et al., 2020; Hu S. et al., 2021) or bio-modified (biosimilar) therapeutics (Xin et al., 2017). These combination therapies can also utilize the protection, stability, and targeting capacity that EVs provide to mediate the delivery of drugs or gene vectors which are otherwise degraded or cleared. Trials *in vivo* have proven the efficacy and feasibility of EV-mediated adeno-associated virus (AAV) and siRNA delivery when uptake levels of the component alone are sub-optimal (Gyorgy et al., 2017; Munagala et al., 2021). Indeed, in *Lhfpl5*−/− hereditary deafness, EV-mediated delivery of AAV was more effective in rescuing hearing than AAV treatment alone (Gyorgy et al., 2017). Additionally, EVs delivering siRNA-*KRAS* were able to successfully silence *KRAS* in murine tumor tissues, reducing tumor size; the same technology also enabled the delivery of plasmids to successfully replace/restore genes (Munagala et al., 2021). Furthermore, the incorporation of drugs and molecular factors into EVs improves their bioavailability and delivery, increasing efficacy of treatment (Aqil et al., 2017; Bellavia et al., 2017; Luan et al., 2017; Liang et al., 2020, 2021). EVs containing curcumin (a natural anti-inflammatory compound) significantly inhibited tumor size when compared with either component alone by increasing curcumin’s bioavailability (Aqil et al., 2017). Indeed, the success of this treatment *in vivo* has led to an active clinical trial investigating the effect of curcumin-containing plant exosomes in the treatment of colon cancer (NCT01294072). While there are few clinical trials using EV-combination therapies (NCT04747574, NCT01294072, and NCT03384433), the pre-clinical success supports the translational potential of this approach.

### Commercial Pursuit of Clinical Translation

Already the revolution of EV therapeutics is underway, with many emerging and established companies now focused on their development and application (Gimona et al., 2017; Zipkin, 2019). Ventures in treatments for dermatological disorders and skin repair (Aegle Therapeutics, XOStem Inc., Exogenus Therapeutics), as well as cancer (Aethlon Medical, Unicyte AG, TAVEC Pharmaceuticals, Puretech Health, EV Therapeutics Inc., Anjarum Biosciences, Codiak Biosciences) and neurological disorders/diseases (Stemcell Medicine Ltd., Puretech Health, Evox Therapeutics, Codiak Biosciences) dominate the commercial EV-based therapeutic landscape. Codiak Biosciences have identified two EV-associated membrane proteins (internal/lumen or external orientated), which they use as scaffolds to link molecules of interest and engineer EVs for therapeutic application; this engineering platform (engEx^TM^) has been utilized to develop exoSTING (EVs enriched in stimulator of interferon genes in the lumen) (Jang et al., 2021), and exoIL12 (Lewis et al., 2021), currently in Phase 1/2 clinical trial (NCT04592484). Significant efforts in other areas include wound healing (RION Health), metabolic disorders (Evox Therapeutics), fibrotic and immunological conditions (Puretech Health), acute respiratory distress syndrome (Direct Biologics), kidney and liver disease (Unicyte AG), and inflammation (Direct Biologics, Cell-Factory BVBA, Puretech Health). Also in development are treatments for specific debilitating and/or life-threatening disorders (VivaZome Therapeutics), retinal disease (ReNeuron), genetic diseases (Anjarium Biosciences), diabetes (Unicyte AG), stroke (ReNeuron), lymphatic disorders (Puretech Health), and cystic fibrosis (OmniSpirant). With EV therapeutics being developed to combat a wide variety of diseases, understanding these membranous entities, their activities, and their engineering are all critical for achieving full therapeutic potential.

## EVs as Nanocarriers of Functional Cargo

Native EVs are cell-derived, lipid-bound nanoparticles (30–1000 nm) that facilitate intercellular communication through targeted delivery of bioactive cargo to alter recipient cell phenotype (Al-Nedawi et al., 2008; Skog et al., 2008; Alvarez-Erviti et al., 2011; Peinado et al., 2012; Kamerkar et al., 2017; Wu et al., 2019; Cocozza et al., 2020; Li et al., 2021b) [reviewed in Vader et al. (2016), Murphy et al. (2019), and Russell et al. (2019)]. EVs are involved in transferring molecular cargo, between animal cells (Valadi et al., 2007; Mittelbrunn et al., 2011; Ono et al., 2019), from parasite to its mammalian host (Buck et al., 2014; Coakley et al., 2017), and from plant to fungal cells (Cai et al., 2018). In addition to their role in communication between cells and organs, EVs are excellent therapeutic delivery systems due to their: (i) ability to protect bioactive cargo, (ii) inherent biocompatibility, (iii) small size and negative surface charge, (iv) ability to cross biological membranes, including blood-brain barrier (BBB), and (v) capacity to target specific cells. The rigid structured membranes [resulting from an abundance of sphingomyelin (Niemela et al., 2004; Saeedimasine et al., 2019; Smith et al., 2020) and cholesterol (Needham and Nunn, 1990; Leftin et al., 2014)] of EVs protect biological macromolecules from degradation [i.e., nucleases (Skog et al., 2008; Cheng et al., 2014; Fernando et al., 2017) and proteases (Haney et al., 2015; Cvjetkovic et al., 2016; Sterzenbach et al., 2017; Zaborowski et al., 2019)] and micro-environmental changes [i.e., pH (Parolini et al., 2009; Aoi and Marunaka, 2014) and osmolarity (Fathali et al., 2017)]. Therefore, compared to soluble factors, EVs more efficiently deliver a complex variety of active biomolecules (Obregon et al., 2009) resulting in greater potency (Mitchell et al., 2019; Cocozza et al., 2020; Gurung et al., 2020). As a result of their cellular origin, EVs are associated with low immunogenicity (Zhu et al., 2017; Sharma et al., 2020) and are capable of interacting with innate immune cells through surface-expressed components (Admyre et al., 2003; Tietjen et al., 2014; Rao et al., 2015; Antes et al., 2018) [e.g., anti-phagocytic signals (Rodriguez et al., 2013; Gordon et al., 2017; Kamerkar et al., 2017; Hsu et al., 2018; Tang Y. et al., 2019; Cordonnier et al., 2020; Daassi et al., 2020) and opsonins (Miksa et al., 2009)]. EV-mediated crosstalk may occur unidirectionally or reciprocally or even *via* systemic communication, during which EVs target to various tissues and organs. Importantly, the EV surfaceome also facilitates inherent targeting properties/tropism (Nakamizo et al., 2005; Kidd et al., 2009; Hoshino et al., 2015; Wu et al., 2020) and direct signal transduction (Nazarenko et al., 2010; Rana et al., 2011; Sobo-Vujanovic et al., 2014; Vinas et al., 2018). Further, EV surface composition is important for traversing biological barriers [e.g., BBB (Haney et al., 2015; Hall et al., 2016; Kojima et al., 2018)] through receptor-mediated transcytosis. The mechanisms by which EVs are internalized by target cells are still poorly understood, often uptake is cell and context dependent. Currently known cellular entry routes of EVs are complex mechanisms ranging through receptor-mediated endocytosis, lipid raft interactions, clathrin interactions, phagocytosis, micropinocytosis and possibly direct fusion (Mathieu et al., 2019). As such, uptake and intracellular mechanisms of trafficking and localization requires more in-depth evaluation exploring subcellular analyses using high-resolution microscopy, subcellular composition studies, or novel live-cell reporters (Chen M. et al., 2018; Sung et al., 2020). In this section, we discuss EVs as carriers of functional cargo and their therapeutically promising characteristics.

### Cell Reprogramming Using EVs

Preclinical development of EVs have focused on their ability to horizontally transfer bioactive components, eliciting transcriptional and translational modulation (Valadi et al., 2007), antigen presentation (Gurnani et al., 2004; Garcia et al., 2016), and functional regulation in recipient cells (Al-Nedawi et al., 2008; Skog et al., 2008; Alvarez-Erviti et al., 2011; Peinado et al., 2012; Kamerkar et al., 2017; Wu et al., 2019; Zhang Q. et al., 2019; Cocozza et al., 2020; Li et al., 2021b). A landmark study demonstrated that embryonic stem cell derived EVs contribute to epigenetic reprogramming of target cells (Ratajczak et al., 2006); EVs were enriched in key players for early pluripotency, hematopoietic stem cells (Scl, HoxB4 and GATA 2), and induced MAPK/Akt phosphorylation. Here, mRNA could be delivered by EVs to target hematopoietic stem/progenitor SKL cells and translated as Oct-4 protein expression. Of note, the biological effects of EVs were perturbed following heat inactivation or pretreatment with RNAse, indicating major involvement of the horizontal transfer of mRNA/proteins by EVs in the observed phenomena. Recently, adipocytes have been shown to release lipid-filled EVs that serve as the primary source of lipid for adipose-resident macrophages (Flaherty et al., 2019). Adipocyte-derived EVs have been described previously, primarily as regulators of inflammation and systemic insulin resistance (Thomou et al., 2017). The uptake of adipocyte EVs facilitated the direct transfer of lipids, and a macrophage-type specific transcriptional activation of lipid metabolism in bone marrow cells., revealing a form of intercellular communication and nutrient exchange with important implications for obesity-associated pathologies (Flaherty et al., 2019). Thus, EVs exert functional effects on recipient cells by modifying their composition through protein, lipid or nucleic acid transfer. This mechanism of action has also been demonstrated in other diseases (i.e., sepsis, fibrosis, cardiomyopathy) where various induced disease models were used to highlight the protective and regenerative roles of EVs, as well as identify the key EV-associated bioactive molecules involved in such functions (Table 2), which provides a strategy to improve the efficacy of EV treatment. In an acute myocardial infarction (AMI) murine model (Gu et al., 2018), EV-mediated transfer of miR-21 protected cardiomyocytes from oxidative stress-induced apoptosis (Xiao J. et al., 2016; Shi et al., 2018) through the inhibition of pro-apoptotic PDCD4 (Xiao J. et al., 2016; Gu et al., 2018) and cell cycle-regulator PTEN (Shi et al., 2018). Treatment of miR-21 mimics *in vitro* and *in vivo* then significantly reduced apoptosis in cardiomyocytes following AMI, and dramatically attenuated infarct size in mouse hearts. As conduits of bioactive molecule transfer, EVs are also exploited as nanocarriers/delivery systems that provides a sustainable mode of miRNA delivery. Transfer of miRNAs from miR-let7c-overexpressing MSCs to renal cells *via* secreted EVs, suppressed the expression of fibrotic genes, thus reducing interstitial collagen production, attenuating renal fibrosis, and improving kidney structure (Wang et al., 2016).

Extracellular vesicles have also been shown to contain and transfer tyrosine kinase receptors (i.e., EGFRvIII) to increase downstream signaling events (MAPK, AKT) and expression of EGFRvIII-regulated genes (*VEGF, Bcl-xL, p27*) involved in tumor vascularization, survival and proliferation (Al-Nedawi et al., 2008). EV-mediated delivery of functional receptors can also be physiological, as demonstrated by the transfer of glucose transporters (GLUT) 1 and 4 between cardiomyocytes and endothelial cells during metabolic stress to increase glucose uptake by endothelial cells and mediate plasma glucose concentration (Garcia et al., 2016). In addition to their functional value, the presence of intact receptors in EVs can facilitate loading of bioactive ligands through their ligand-receptor interaction, increasing the efficacy of EV function on recipient cells. As a cerebral hypoxia/ischemia treatment, EV-bound transferrin receptor was exploited to load apotransferrin into EVs, which, when administered to animal demyelination models, conferred neuroprotection and reduced white matter damage, neuronal loss, and astrogliosis through apotransferrin-activated differentiation of oligodendroglial cells in the brain (Mattera et al., 2020).

This protective effect of EVs have also been shown to rescue adverse side-effects following chemotherapy-induced damage. EVs derived from amniotic fluid stem cells enriched in pro-survival miR-146a and miR-10a were directly injected into murine ovaries, where they promoted the survival and elevated the apoptotic resistance of granulosa cells in mice undergoing chemotherapy; side effects can otherwise cause premature ovarian failure and aberrant fertility (Xiao G. Y. et al., 2016). This is attributed to the apoptosis- and inflammation-related downstream targets of EV-associated and transferred miR-146a and miR-10a, including IRAK1 and TRAF6, and BIM respectively (Xiao G. Y. et al., 2016). However, if these anti-apoptotic EVs are trafficked to off-target sites (i.e., tumors), they can significantly reduce the sensitivity of tumor cells to chemotherapy treatment, as demonstrated by EV-mediated transfer of miR-19b (Gu Y. Y. et al., 2019). Therefore, stringent regulation of EV-associated bioactive cargo and components of interest is critical in developing EVs for therapeutic application.

For further streamlining of functional/therapeutic effect, EV donor cell source is a critical factor for consideration, particularly in the field of regenerative medicine, where MSCs, with unique multipotent differentiation potential (Sasaki et al., 2008; Sarugaser et al., 2009; Dos Santos et al., 2019), are expansively explored in various pathologies and regenerative applications. Importantly, MSC-derived EVs have been shown to alleviate CNS-associated disorders/injuries, including subcortical stroke [i.e., through axonal sprouting, tract connectivity, remyelination and oligodendrogenesis (Otero-Ortega et al., 2017)], and, cerebral ischemic-reperfusion injury [i.e., by conferring neuroprotection through modulation of M1-M2 microglia toward an anti-inflammatory phenotype (Liu et al., 2021)]. They are also an avenue for treatment of physical ailments, such as osteoarthritis [i.e., by attenuating inflammation and regulating extracellular matrix synthesis/degradation (Cosenza et al., 2017; Wang et al., 2017; Zhang S. et al., 2019)], skeletal muscle injury [i.e., through promotion of skeletal muscle cell proliferation, differentiation and migration (Mitchell et al., 2019)], and ageing [i.e., by ameliorating dermal fibroblast senescence and promoting skin rejuvenation (Zhao et al., 2021)]. In models for cardiac injury, MSC-derived EVs have been shown to promote proliferation and differentiation of epicardial-derived cells and improve cardiac morphology (Arslan et al., 2013; Zhu et al., 2021b). In fact, improvements for sustained administration of MSC-EVs have been made to enhance their effects at sites of cardiac (Chen C. W. et al., 2018), spinal cord (Li L. et al., 2020), or hepatic injury (Mardpour et al., 2019), by loading them into biocompatible hydrogels which are then administered to target sites.

For therapeutic development, it must be noted that EV composition and release is influenced by environmental and signaling changes occurring in donor cells {i.e., hypoxia, stress [metabolic (Fan et al., 2020), heat, oxidative], infection (Pegtel et al., 2010) and cell activation (Gao et al., 2019, 2020)} as a mechanism of homeostatic maintenance (Takahashi et al., 2017) [reviewed in Desdin-Mico and Mittelbrunn (2017)], thus, EV donor cell culture conditions may be modified accordingly to suit various clinical applications. For instance, MSCs under high oxygen level-induced oxidative stress release depolarized mitochondria to remove accumulated reactive oxygen species (ROS) generated as a result of oxidative phosphorylation (Phinney et al., 2015). The EV-associated mitochondria are transferred to and metabolically reprogram recipient cells with potent regenerative effects, as observed in cardiomyopathy (Plotnikov et al., 2008; Ikeda et al., 2021), and renal (Plotnikov et al., 2010) and pulmonary (Islam et al., 2012) injury. Another key, yet not fully explored consideration in this study, is the synergistic function of EV cargo. For example, in a pulmonary silicosis-induced murine model, MSC-derived EVs transfer both mitochondria and anti-inflammatory and immunomodulatory miR-451 to macrophages; increasing their metabolic state while inhibiting their activation and protecting them from excessive inflammation caused by accumulated mitochondrial ROS (Phinney et al., 2015). The synergistic function performed in this study by EV-associated mitochondria and anti-inflammatory miRNA is therefore a well-balanced one, highlighting a need for in-depth EV composition characterization during their development as therapeutics. Modifying protein cargo loading into EVs can be manipulated in donor cells directly and exploiting endogenous protein sorting mechanisms. Ubiquitin-like 3 (UBL3) was identified as the protein sorting target which can interact with and post-translationally modify cargo proteins (i.e., Ras and tubulin) to enhance their loading into EVs (Ageta et al., 2018). Through donor cell transfection of mutated, oncogenic *RasG12V*, a binding partner of UBL3, this study demonstrated effective loading into released EVs and subsequent downstream signaling activation in target cells (Ageta et al., 2018). Development of loading proteins into EVs from the cytosol in a free form has been shown *via* optically reversible protein-protein interactions; EXPLORs (Yim et al., 2016). This approach is based on the selective interaction of cargo protein and tetraspanin CD9, allowing cargo proteins to be freely localized in the recipient cells. On the other hand, loading of specific miRNA can be achieved by exploiting the short sequence motifs over-represented in miRNAs commonly enriched in EVs, and the sumoylation of their binding partner hnRNPA2B1, which is required to facilitate miRNA sorting into EVs (Villarroya-Beltri et al., 2013). Other loading RNA-based methods using RNA binding proteins include EXOsomal transfer into cells (EXOtic) using the interaction between C/D box RNA structure and L7Ae ribosomal protein fused with tetraspanin CD63 (Kojima et al., 2018), and conjugated mRNA sequence with interaction between *trans*-activating response (TAR) sequence and *trans*-activator of transcription (Tat) protein (with membrane protein ARMMs) (Wang Q. et al., 2018), to load specific mRNAs into EVs. Further, this latter study demonstrated a highly versatile platform for packaging and intracellular delivery of therapeutic macromolecules, including protein, RNAs, and the genome-editing CRISPR-Cas9/guide RNA complex (Wang Q. et al., 2018). While the molecular mechanisms of cargo sorting into EVs are still being understood, it may be useful to consider the above characteristics of target molecules and their compatibility with endogenous donor cell cargo sorting machinery to improve their loading efficiency and consistency for EV-based therapeutic development.

### Targeted Delivery of EVs

Specific components present in the EV surfaceome (selections of proteins presented on the external side of the lipid bilayer), allow for preferential uptake by specific cells, tissues, and organs (Peinado et al., 2012; Oksvold et al., 2014; Costa-Silva et al., 2015; Hoshino et al., 2015; Belov et al., 2016; Rodrigues et al., 2019; Wu et al., 2020). Although this capacity for tropism is supported by various reports, our understanding of the complexities behind this intrinsic targeting in native EVs remains incomplete, though they are widely attributed to their biological origin. Like their progenitor cells, platelet-derived EVs have an intrinsic affinity with inflammatory sites and atherosclerotic plaques, binding to activated/inflamed vascular walls and plaques through various receptors including CD40L (Mach et al., 1997; Lievens et al., 2010), glycoproteins Iba and aIIb (Guo et al., 2015; Pawlowski et al., 2017), and P-selectin (Dinkla et al., 2016; Pawlowski et al., 2017). Thus, platelet-derived EVs hold great potential to be exploited as nanocarriers of anti-inflammatory reagents to ameliorate inflammatory diseases including pneumonia (Ma et al., 2020) and atherosclerosis (Song et al., 2019). EV homing to specific organs also play roles in disease progression, such as those derived from pancreatic ductal adenocarcinoma cells (PDAC) (a cancer commonly associated with liver metastasis), which demonstrated natural homing to the liver upon *in vivo* administration, where they were selectively taken up by Kupffer cells (Costa-Silva et al., 2015). Though the molecular mechanism behind this liver tropism is unknown, the current hypothesis is that EVs express surface components to facilitate a specific interaction with target cells. Indeed, EVs derived from tumors with metastatic organ tropism (lung, liver, or brain) express different integrin complexes; lung-tropic: α6β4 and α6β1, liver-tropic: αvβ5, brain-tropic: ITGβ3 (Hoshino et al., 2015). EVs containing these integrin complexes bind to S100-A4-positive fibroblasts and surfactant protein C-positive epithelial cells in the lungs, Kupffer cells in the liver, or CD31-positive endothelial cells in the brain (Hoshino et al., 2015), respectively. Lung-tropism is also influenced by non-integrin proteins, with genetic knockdown of SLCO2A1, CD13, and CLIC1 in cervical adenocarcinoma cells (HCA1) resulting in the decreased accumulation of EVs in lung tissue (Wu et al., 2020). EVs’ tropism to particular organs and cells may also be mediated by surface ligands that interact with cognate receptors on recipient cells to induce binding and subsequent uptake. This ligand-receptor interaction (i.e., EV chemokine receptor CXCR4 to recipient cell-expressed chemokine SDF-1α) mediated targeting of endothelial colony forming cell-derived EVs to the kidney, thus preventing kidney injury and neutrophil infiltration following ischemic injury through the transfer of PTEN-inhibitor miR-486-5p (Vinas et al., 2018).

Other surface-expressed molecules that mediate EV-cell interaction include glycosphingolipid glycan groups, which are present on neuroblastoma-derived EVs, facilitating their binding to and clearance of β-amyloid aggregates in the brain (Yuyama et al., 2014). Their ability to target the brain highlights the potential of EV-based treatments for brain cancer and Alzheimer’s disease. Other mechanisms for brain-tropism also exist, such as the natural homing capacity of EVs derived from specialized immune cells in the central nervous system (CNS) (i.e., microglia) to the brain, where they reduced multiple sclerosis-associated inflammation in myeloid cells and astrocytes through the transfer of anti-inflammatory cytokine IL-4 (Casella et al., 2018). These studies highlight the natural ability of EVs to transverse the BBB, an ability mediated in-part by surface components (i.e., heparan sulfate proteoglycans, mannose 6-phosphate receptor, CD46, integrin complexes αVβ6 and αVβ3, and endothelial- and leukocyte-associated transmembrane protein ICAM-1) which allow receptor-mediated transcytosis (Banks et al., 2020; Joshi and Zuhorn, 2021); a transcellular route allowing EVs to traverse brain endothelial capillary cells toward brain parenchyma. Further characterization of surface components which possess tropic-capacity will allow for refinement and selection of EV therapeutics.

### Biodistribution and Clearance of Native EVs

As mediators of inter-organ communication, EVs must evade immune clearance for as long as possible to remain in circulation prior to cellular uptake (Smyth et al., 2015; Charoenviriyakul et al., 2017). The mononuclear phagocyte system (MPS) [previously termed reticuloendothelial system (RES)] encompasses monocytes, macrophages, and other cells present in liver, spleen, and lungs, and contributes to EV sequestration and clearance (Rao et al., 2015; Smyth et al., 2015). Indeed, following *in vivo* administration, EVs accumulate in the liver, spleen and/or lung; an occurrence widely observed in EVs derived from dendritic (Wei et al., 2017), MSCs (Grange et al., 2014), myoblasts (Wiklander et al., 2015; Charoenviriyakul et al., 2017), kidney (Lai et al., 2014), glial (Lai et al., 2014), melanoma (Peinado et al., 2012; Takahashi et al., 2013; Imai et al., 2015; Charoenviriyakul et al., 2017), macrophages (Charoenviriyakul et al., 2017), and placental (Tong et al., 2017; Nguyen et al., 2021) cells. With phagocytosis central to clearance, EVs avoid engulfment through surface presentation of anti-phagocytic signals including immunomodulatory receptors, most commonly CD47 (Chao et al., 2012; Rodriguez et al., 2013; Kaur et al., 2014; Kamerkar et al., 2017; Tang Y. et al., 2019), PD-L1 (Gordon et al., 2017; Hsu et al., 2018; Cordonnier et al., 2020; Daassi et al., 2020), CD24 (Barkal et al., 2019), CD31 (Brown et al., 2002), and CD44 (Vachon et al., 2007; Amash et al., 2016), that act as “don’t eat me” signals to phagocytic cells, potentially prolonging their EV half-life in circulation.

Conversely, internalization of EVs ensures the delivery of EV content into target cells, allowing them to exert their effector functions. Phosphatidylserine (PS) is a lipid located on the outer leaflet of EV membranes, forming lipid-receptor interactions [e.g., with immune regulatory receptor TIM4 (Tietjen et al., 2014)] to facilitate EV engulfment and internalization by recipient cells (Lindemann, 1989; Fadok et al., 2000; Gurnani et al., 2004). EVs also express opsonins [e.g., MFGE8 (Dasgupta et al., 2009; Miksa et al., 2009; Casella et al., 2018), β2-glycoprotein-1 (Abdel-Monem et al., 2010), developmental endothelial locus-1 (Dasgupta et al., 2012)] that facilitate their internalization through PS-dependent phagocytosis. Indeed, increasing the expression of MFGE8 on microglia-derived EVs elevated their uptake by macrophages and microglial cells in the CNS, facilitating the transfer of EV-loaded anti-inflammatory cytokine (i.e., IL-4) to significantly reduce clinical symptoms of neuro-inflammatory multiple sclerosis, and experimental autoimmune encephalomyelitis (EAE) in a murine model (Casella et al., 2018). Other factors that influence EV biodistribution and uptake *in vivo* is the microenvironment from which they are released. Tumor-derived EVs can bind to soluble secreted cytokines and chemokines (i.e., CCL2 and IL-6) in the tumor microenvironment through their surface-expressed glycosaminoglycan (GAG) side chains of proteoglycans, significantly increase their uptake in the liver, spleen, and lung (Lima et al., 2021). Through a similar mechanism, EVs expressing GAGs released by glioblastoma bind to, and are decorated with, chemokine ligand CCL18, facilitating their interaction with cognate receptor CCR8 on recipient glioblastoma cells (Berenguer et al., 2018) to increase uptake and induce a proliferative phenotype (Berenguer et al., 2018). Indeed, pharmacological inhibition of this interaction (*via* CCR8 inhibitor, R243) completely blocked EV-induced tumor growth, thus neutralizing EV-induced phenotypic remodeling (Berenguer et al., 2018).

Despite these insights into EV biodistribution and clearance, attempts to comprehensively define the pharmacokinetics of EVs *in vivo* have remained inconsistent, with results ranging from their rapid clearance within 2–4 min (Takahashi et al., 2013) (as monitored by fluorescence imaging) up to 7 days, with DiR-labeled EVs localized to the liver and spleen a week following administration (Liu H. et al., 2016). This variation may be resultant of EV labeling with lipophilic dyes, which have been found to remain in the system long after EVs have been degraded or recycled (Takov et al., 2017). Regardless of these uncertainties, EVs possess important qualities—highlighting them as promising modalities for therapeutic applications. Further development toward EV-based clinical application involves their engineering/modification to reduce their immune clearance and prolong their half-life in circulation, improve their biodistribution and tropism to sites of interest, and, enhance their functionality on recipient cells, tissues, and organs.

## Engineered EVs

Native EVs offer unique advantages for cellular regulation and the efficient delivery of therapeutic payloads. However, several studies have highlighted the intrinsic limitations of native EVs including long-term maintenance of parental cell culture with minimal metabolic/phenotypic variation (Lambshead et al., 2018; Cherian et al., 2020; Escude Martinez de Castilla et al., 2021), bio-distribution (i.e., organ targeting) (Escude Martinez de Castilla et al., 2021; Lazaro-Ibanez et al., 2021; Ullah et al., 2021; Witwer and Wolfram, 2021), clearance rates [complement and immune systems (RES-MPS system)] (Wiklander et al., 2015; Ha et al., 2016; Kwon, 2020; Lara et al., 2020; Buschmann et al., 2021; Escude Martinez de Castilla et al., 2021; Lazaro-Ibanez et al., 2021), and crucially, difficulties associated with large scale generation (i.e., processing times, variable potency between batches, good manufacturing practice; GMP) (Whitford and Guterstam, 2019; Cherian et al., 2020; Buschmann et al., 2021; Escude Martinez de Castilla et al., 2021; Ullah et al., 2021; Witwer and Wolfram, 2021). Moreover, the regulatory machinery of EVs and their distinct subtypes associated with production and cellular uptake remains largely unknown. Engineering therapeutics based on native EVs offers an alternative approach, employing their advantages while bypassing limitations. As such, the engineering of EVs for therapeutic application is a field undergoing rapid development with applications for regeneration/repair, immune disorders, wound healing and cancer (Commisso et al., 2013; Kordelas et al., 2014; Xitong and Xiaorong, 2016; Gyorgy et al., 2017; Wu et al., 2019; Li L. et al., 2020; Hu S. et al., 2021; Shi et al., 2021). Here, we discuss several strategies recently developed to address such limitations through modification or engineering of EVs including: (i) generation of mimetic EVs/nanovesicles (M-NVs), EV synthetics (synEVs), and EV hybrids (hEVs), (ii) improvement of targeting of native EVs and (iii) customized cargo loading (into native and engineered EVs) to enhance their functional properties (Table 3).

### Engineering Alternatives to EVs

M-NVs simulate the biophysical properties of native EVs, including size (50–200 nm), which has been reported to influence half-life in circulation (i.e., nanoparticles smaller than 200 nm are able to evade RES uptake, and nanoparticles larger than 30 nm to avoid rapid renal elimination) (Ha et al., 2016; Murphy et al., 2019; Lara et al., 2020; Buschmann et al., 2021; Dooley et al., 2021; Escude Martinez de Castilla et al., 2021; Lazaro-Ibanez et al., 2021; Witwer and Wolfram, 2021). M-NVs can be generated by either top-down (extruding parental cells into nano-sized fragments) to obtain biological M-NVs, or bottom-up methods (selecting cargo and capsule materials i.e., liposomes or polymer nanoparticles) to obtain chemically-defined synEVs (Nasiri Kenari et al., 2020). Top-down M-NVs are generated by serial extrusion, ultracentrifugation, or pressure-based microfluidic approaches [reviewed in Mentkowski et al. (2018)]. Mechanical extrusion is achieved by forcing the cell suspension to pass through membranes of different pore size to cause cell disruption (Jang et al., 2013). After extrusion of parental cells, the membrane fragments form membrane-derived vesicles due to their physicochemical properties, engulfing the cellular components in suspension and generating mimetic EVs (Jo et al., 2014b). Following this method, M-NVs generated from macrophages through serial extrusion with concurrent loading of catalase (added to the cell suspension before extrusion) demonstrated elevated neuroprotective activity while increasing the yield ∼100-fold, compared to native EVs (Haney et al., 2015).

MN-Vs can also be generated from MIN6 pancreatic β-cells by serial extrusion i.e., five passages through 10, 5 and 1 μm polycarbonate membrane filters using a mini extruder (Oh et al., 2015). In parallel, bone marrow MSCs isolated from femurs and tibias of BALB/c mice were embedded in Matrigel and implanted subcutaneously into NSG mice (NOD.Cg-PrkdcscidIl2rgtm1Wjl/SzJ). Into these subcutaneous patches, 10 μg/injection of MIN6 mimetics were administered for a total of four injections in 10 days. The authors demonstrated that the MSCs imbedded in Matrigel formed islet-like clusters with extensive capillary networks as a cause of M-NV administration, and that these were able to maintain the glucose levels of the mice for over 60 days (Oh et al., 2015). This work provided a key insight of the effectiveness of mimetics *in vivo* as delivery and signaling systems. To understand composition of M-NVs, Hill and collaborators (Nasiri Kenari et al., 2019) performed proteomic profiling of M-NVs and demonstrated their distinct composition from exosomes and parental cells. Although M-NVs shared many similarities with native EVs (physical attributes, key protein EV markers, proteins that overrepresented the original cell), differences were observed in protein post-translational modifications, specifically phosphorylation, ubiquitination, and thiophosphorylation. This raises an important consideration of using M-NVs as an alternative nanocarrier when spontaneous endosomal sorting of therapeutics is limited or when modulation of donor cells influences native EV generation. An important consideration of M-NV generation is that despite the 100 to 150-fold increase in yield, this method still relies in parental cells as raw materials which requires the surveillance of their long-term maintenance (genome stability, passage number, cell culture technologies) and culture conditions/media type and potential influence of non-model EV source [i.e., bovine-derived (Eitan et al., 2015; Lehrich et al., 2021; Pham et al., 2021)].

A different stream of research to solve upscaling as a key limitation for native EVs’ and M-NVs’ pharmaceutical use, focuses on the generation of bottom-up particles denominated synEVs. synEVs have demonstrated a high efficacy and high scalability as drug and vaccine-based delivery systems (Park et al., 2021). Generation of liposomes include extrusion over membrane filters [analogous to mimetic generation (Cao et al., 2016; Nele et al., 2019; Shah et al., 2019)], and hydrophilic microchannels [microfluidic systems (Shah et al., 2019; Kotoucek et al., 2020)]. The development of synEVs has gained immense interest due to COVID-19 vaccine research, including mRNA encapsulated in liposomes by Moderna/NIAID, BioNTech/Pfizer, Arcturus/Duke-NUS, PLA/Walvax Biotech, Imperial College London, and CureVac AG (Park et al., 2021). These synEVs (in particular Moderna/NIAID and Pfizer non-viral vaccines) have shown to be a highly efficient system for delivery and immunoregulatory response (Park et al., 2021). Furthermore, the methods for generation and encapsulation of cargo (i.e., mRNA) into synEVs has demonstrated high efficiency and yield; >72% encapsulation rates (Hassett et al., 2019). Although different approaches for generating synEVs with vaccine applications have been employed, in all cases their lipid composition provides key therapeutic advantages, including ability to encapsulate and condensate mRNA, promote delivery to cytosol by increasing cellular uptake (due to their composition compatible with biological membranes i.e., PEGylated lipids, cholesterol and cationic or ionizable lipids), protect mRNA (or any other cargo) from degradation in extracellular spaces, and their components are easily manufactured with GMP in a large scale. The latter demonstrates synEVs represent a unique advantage in combining with production of native EVs, despite issues with their targeted delivery. Unlike cell-derived EVs, synEVs lack targeting and recognition molecules, therefore, their synthesis has been coupled with different functionalization techniques such as bioconjugation (Smyth et al., 2015; Lim et al., 2021) [reviewed in Murphy et al. (2019), Rayamajhi and Aryal (2020), Salmond and Williams (2021), Sharma et al. (2020), and Takayama et al. (2019)] and cargo loading (Haney et al., 2015, 2019) [reviewed in Luan et al. (2017), Nasiri Kenari et al. (2020), Roberts et al. (2020), and Sterzenbach et al. (2017)] to obtain use-specific synEVs (Garcia-Manrique et al., 2018), as well as their fusion with EVs (native or mimetics) to generate EV hybrids (hEVs).

hEVs are a recently developed method to generate vesicles—they comprise native EV components and synthetic liposomes (Gangadaran and Ahn, 2020). Hybrids could be a more effective alternate to both EVs and liposomes as drug delivery systems by combining the advantages of loading versatility (diverse molecular cargo), targeting capabilities (native EV tropism), and stability (structure stabilization, cargo protection, handling stability) [reviewed in Ou et al. (2021)]. Moreover, hEVs can be generated by different methods such as extrusion, sonication, co-incubation or freeze/thaw cycles, which makes them convenient for clinical development and diverse applications (Gangadaran and Ahn, 2020). Freeze/thaw cycle method was used in combination to surface modification techniques to fuse EVs expressing a specific surface protein produced by macrophages and different cancer cell lines (mouse fibroblast sarcoma-derived CMS7-wt, CMS7-HE, and Raw 264.7 macrophages) with liposomes (Sato et al., 2016). Hybrids have also been generated by sonication of an aqueous suspension of macrophage-derived EVs and L-α-phosphatidylcholine/Cholesterol liposomes (Rayamajhi et al., 2019). The resulting hybrids are a promising platform for tumor-targeted drug delivery, releasing Doxorubicin predominantly to macrophages, osteosarcoma cells and breast cancer cells (compared to normal fibroblasts) *in vitro*, demonstrating these hEVs have preferential targeting to parental cells and tumor cells (Rayamajhi et al., 2019). Furthermore, a variant of hEVs using native EV sources [bone marrow MSC-derived EVs (purified from conditioned media) and platelet mimetics (generated by a combination of freeze/thaw and consecutive sonication)], were generated by co-extrusion and administered to mice (100 μg/mice, once a week for 4 weeks), demonstrated increased targeting and pro-angiogenic activity in a mouse model of myocardial infarction in mice (Li et al., 2021c). Here, tropism of platelets toward activated endothelium (which occurs during injury or stress) was inherited by the resulting hybrids enhancing their targeting and accumulation capabilities to the myocardium (compared to native MSC-derived EVs) allowing the targeted delivery of reparative cargo despite systemic administration.

### Improved Targeting: Genome Engineering and Surface Functionalization

Modifying EVs to improve their specific delivery is a key requirement in therapeutic applications. Several engineering approaches have been developed and applied to EVs. Click chemistry, genetic modification, and glycoengineering have proven to be highly efficient methods to increase site-specific retention, thus reducing off target effects (Smyth et al., 2014; Williams et al., 2018; Rayamajhi and Aryal, 2020). EV composition and function are influenced by the cell source, a characteristic which can be exploited during EV-based therapeutic development (Kim et al., 2020). Many technologies can modify progenitor cells to alter the functional capacity of their derived EVs; one of which is genome engineering, which involves either the knockout [i.e., siRNA, CRISPR (Horodecka and Duchler, 2021)] or overexpression [i.e., Lentiviral/Adenoviral, plasmid, or nucleic acids (siRNA, miRNA, anti-miR) transfections (Li et al., 2013; Zhang et al., 2014; Chen S. et al., 2019; Escude Martinez de Castilla et al., 2021)] of genes (Shi et al., 2020). The composition of EVs released from these modified cells are therefore customized, heavily influencing recipient cell function [i.e., immunomodulatory (Trivedi et al., 2016; Gomez-Ferrer et al., 2021), pro-angiogenic (Zuo et al., 2019; Zhang L. et al., 2021), anti-apoptotic (Wen et al., 2020) or anti-cancer (Kim R. et al., 2018)]. Thus, these strategies are applicable to a range of pathologies, including cardiovascular disease, tissue repair/regeneration, cancer, and immunological disorders. However, continuous improvements are required for these techniques due to their low mutation efficiency and potential off-target sequence error (CRISPR-Cas9) (Siddique, 2016) or low efficiency and variable expression levels by transfected cells (transfection) (Di Blasi et al., 2021) (which may influence the biogenesis/yield/content/function of the produced EVs (Carli et al., 2021) or parental cell viability or proliferation (Lambshead et al., 2018; Cherian et al., 2020; Escude Martinez de Castilla et al., 2021).

In this context, genetic manipulation strategies [e.g., plasmid transfection (Cho et al., 2018; Hong et al., 2019; Shi et al., 2020; Feng et al., 2021), pDisplay vector transfection (Ohno et al., 2013), retroviral transfection (Fan et al., 2013)] pre-EV isolation have been shown to modify the surface of EVs to enable site-specific delivery (Wan et al., 2018) (Table 3). For instance, overexpression of folate receptor α (FRα) on EV surface facilitated their specific binding to the brain parenchyma, crossing the BBB, demonstrating a mechanism of brain-specific drug delivery *in vivo* after an intraventricular injection (Grapp et al., 2013). Furthermore, genome engineering of cardiosphere-derived cells (CDC) using lentiviral particles to express LAMP2B fused to a cardiomyocyte specific peptide (CMP; WLSEAGPVVTVRALRGTGSW) generated EVs displaying LAMP2B-CMP on their surface, increasing retention time and improving targeted delivery to the heart (Mentkowski and Lang, 2019). Moreover, modification of mouse dendritic cells to express LAMP2B fused to the neuron specific RVG peptide was shown to target EVs with siRNA-BACE1 to neuronal cells (Alvarez-Erviti et al., 2011). The therapeutic potential of this system was demonstrated by mRNA (60%) and protein (62%) knockdown of BACE1, a therapeutic target in Alzheimer’s disease. This strategy was also employed to modify BMSCs for the generation of EVs expressing LAMP2B coupled with ischemic myocardium-targeting peptide (IMTP; CSTSMLKAC), which caused a significant increase of EV accumulation in cardiac tissue when compared to peptide sequence (non-targeted) controls (Antes et al., 2018; Wang X. et al., 2018). Altogether, these studies highlight the capacity of genome engineering to generate surface modifications that enhance EV targeting capabilities. On the other hand, off-target errors during genome editing complicate validation and are time consuming/expensive, thus would impede upscaling. It was also observed that despite their targeting capabilities, engineered EVs accumulate in non-targeted tissue/organs, including liver and kidney (Wang X. et al., 2018); determination of the EVs’ effects at these sites and an improved understanding of EV-site interaction are required for clinical applications. Engineering methods can also be combined to refine EV-based therapeutics. Decoy mimetic EVs have been generated through mechanical extrusion of genetically engineered parental-cells overexpressing surface receptor ACE2 (Rao et al., 2020). These mimetic EVs were fused with human myeloid mononuclear THP-1 mimetics, to generate hEVs. Upon administration to a murine model of acute pneumonia, these hEVs suppressed immune disorder and decreased lung injury (Rao et al., 2020).

A different approach for surface engineering is modification post-EV isolation. These modifications can be performed through glycoengineering [attachment of glycans to proteins by generation of covalent bonds (Williams et al., 2018; Williams C. et al., 2019; Sharma et al., 2020; Della Rosa et al., 2021; Lim et al., 2021; Martins et al., 2021)] and click chemistry [reactions which involve conjugation of molecules in a modular fashion, for example, the bio-orthogonal copper-free azide alkyne cyclo-addition (Smyth et al., 2014; Ouyang et al., 2018; Murphy et al., 2019; Takayama et al., 2019)], and are used for targeting (Kooijmans et al., 2016) and cloaking strategies (Kooijmans et al., 2016; Suk et al., 2016), also known as “surface functionalization” (Rayamajhi and Aryal, 2020). For example, glycoengineering of anti-EGFR nanobody to the phosphatidylserine (PS) of EVs derived from blood, or Neuro2A cells, promotes their uptake by EGFR^+^ cells in a dose-dependent manner and decreases their non-specific binding to other cells (Suk et al., 2016). Further, this modification resulted in an increased circulation time of >60 min for engineered EVs in comparison to 10 min for native EVs (Suk et al., 2016). Together, these results provide an effective strategy for cloaking and targeting using glycoengineering.

A combination of glycoengineering and click chemistry has been employed as a highly- efficient and specific approach to modify the EV surface through irreversible bioconjugation (Smyth et al., 2014; Takayama et al., 2019). This allows EV surface functionalization for delivery of small molecules, large biomacromolecules, and polymers without altering particle size or function. Murine MSC-derived EVs were modified by copper-free click chemistry to generate an Ale-EVs system (EVs coupled with Alendronate, a medication used for osteoporosis) administered to an *in vivo* model of ovariectomy (OVX)-induced osteoporosis (Wang Y. et al., 2020). These Ale-EVs had an affinity to bone tissue and promoted regeneration under osteoporotic conditions with low toxicity. Similar methods, including genetic modification of parental cells and/or post-EV isolation methods can also be used to enrich surface-ligands on EVs, which can then induce/inhibit signaling or target specific recipient cells for delivery (Jafari et al., 2020). As a direct approach to modify the surface of MSC-derived EVs by click chemistry conjugation, c(RGDyK) peptide which exhibits affinity to integrin αvβ3 and expressed in reactive cerebral vascular endothelial cells after ischemia, was used for improved delivery (11-fold tropism to the lesion region of ischemic brain) and treatment of stroke (Tian et al., 2018). Additionally, surface modifications using click chemistry can be used to chemoenzymatically label EVs to visualize cellular uptake in real time. EVs isolated from conditioned media of human breast cancer MCF-7 cells were purified and an alkyne group (click chemistry target) substituted for the choline group from native EV phosphatidylcholine by an enzymatic reaction using phospholipase D (Jiang et al., 2020). Using the alkyne-azide click chemistry, fluorescent Cy5 dye was covalently fused and used to track cellular internalization in real time using fluorescent confocal microscopy *in vitro* (Jiang et al., 2020). Furthermore, the surface modification did not alter EV size (compared with native EVs by nanoparticle tracking analysis) (Jiang et al., 2020). This study provides an efficient method for tracking EVs that could be applied to monitor biodistribution, targeting and uptake of engineered EVs.

### Cargo Loading Strategies

In addition to cargo loading *via* surface conjugation (Wang Y. et al., 2020), other strategies used to load cargo into EVs include parental cell modification, passive diffusion, and active loading. Overexpression of components in parental cells is an effective way to increase abundance in resulting EVs for greater function. Transfection of MSCs with miR-133b resulted in accumulation (∼2.5-fold higher levels) of miR-133b in EVs (when compared to EVs from non-transfected MSCs), which subsequently improved functional recovery, reduced lesion volume, and increased neuron survival in an *in vivo* model of spinal cord injury (Li et al., 2018). Several other studies have modified either parental cells (pre-EV isolation) or EVs directly (post-EV isolation) for improved therapeutic response [reviewed in Lara et al. (2020), Nagelkerke et al. (2021), Ullah et al. (2021), and Witwer and Wolfram (2021)] such as in cancer (Zhang et al., 2014), neurodegeneration and Parkinson’s disease (Haney et al., 2015), and kidney fibrosis (Wang et al., 2016; Tang T. T. et al., 2019).

An alternative to modifying parental cells is the direct incorporation of specific molecules (i.e., miRNA, siRNA, protein, lipids, drugs) into EVs through passive or active methods (Luan et al., 2017; Sterzenbach et al., 2017; Nasiri Kenari et al., 2020; Nazimek and Bryniarski, 2020). Incubation of EVs with active components is a passive method of loading that involves the diffusion of drugs or molecules with varied encapsulation efficiency through the EV membrane. This approach has facilitated loading of siRNAs, miRNAs, proteins (i.e., catalase; an antioxidative enzyme, Parkinson’s disease treatment) (Haney et al., 2015), and anti-cancer drugs [i.e., Paclitaxel (Saari et al., 2015; Kim et al., 2016), Doxorubicin (Smyth et al., 2015), Imatinib (Bellavia et al., 2017)] into EVs for delivery. The loading efficiency is dependent on incubation time, cargo concentration and physicochemical properties of the cargo (i.e., solubility, surface area polarity, lipophilicity, hydrophobicity) (Liu C. et al., 2016; Luan et al., 2017; Kim et al., 2020). To address this variability, active loading methods have been implemented (co-incubation with membrane permeabilizers, i.e., saponin) to improve loading efficiency of specific molecules by up to 11-fold (Fuhrmann et al., 2015). This involves temporary and controlled disruption of EV membrane, usually accomplished by sonication, electroporation, membrane permeabilizers, freeze-thaw cycles, or cell extrusion (Table 3) to allow entry of cargo into EVs.

Sonication of EVs (using sonic waves) for therapeutic cargo loading is a widely used method for various clinical applications. Using this technique, macrophage-derived EVs were successfully loaded with Doxorubicin and Paclitaxel, allowing resulting vesicles to target cancer cells and inhibit tumor growth. These modified EVs are an attractive therapy for pulmonary metastases and triple negative breast cancer in murine models (Kim M. S. et al., 2018; Haney et al., 2020). Importantly, these studies observed that pH, temperature, and sonication configuration (time, power, probe, or water bath) affected loading efficiency, and required optimization (dependent on the cargo properties). Using an electrical pulse to temporarily disrupt the EV membrane, electroporation is a widely used method for loading small molecule drugs and nucleic acids into EVs to treat Alzheimer’s disease (Alvarez-Erviti et al., 2011), and breast (Ohno et al., 2013), lung (Takenaka et al., 2019), and ovarian (Kobayashi et al., 2020) cancers. The loading efficiency of nucleic acids (i.e., siRNA) through electroporation ranges between 15 and 20%, demonstrating a robust method for cargo loading (Jhan et al., 2020).

As an alternative to physical membrane disruption, incubation of EVs with chemical membrane permeabilizers also allows efficient loading of therapeutic cargo into EVs. Incubation of EVs with the detergent-like molecule saponin has been used to incorporate antioxidant catalase and the enzyme tripeptidyl peptidase-1 (TPP1) into bone marrow macrophage-derived EVs (up to 6.3 μg/10^11^ particles for catalase, or 50 μg/10^11^ particles for TPP1 were incorporated) (Haney et al., 2015, 2019). The loaded EVs significantly inhibit neurodegeneration and neuroinflammation in mouse models of Parkinson’s disease (Haney et al., 2015) and Batten disease (Haney et al., 2019). Other techniques used for EV cargo loading include multiple freeze-thaw cycles (Kalani et al., 2016), and serial extrusion of cell suspension through decreasing pore sizes in a buffer containing cargo of interest (Kim H. Y. et al., 2018). Studies comparing these processes highlight serial extrusion as the most efficient method for cargo loading, resulting in stable EVs with elevated functional effects (Fuhrmann et al., 2015; Haney et al., 2015; Kim et al., 2016; Le Saux et al., 2020). In the context of EV-based therapeutics, all cargo loading methods have intrinsic advantages and disadvantages that must be considered (Table 3). The methods applied should be modified according to the clinical/disease/model context (i.e., model and tissues of interest, biological/clinical question). Altogether, these advances in EV engineering provide highly customizable and combinatorial techniques to overcome current limitations of native EV as therapeutics, thus improving delivery, efficacy, and function. These advancements have allowed rapid development of EV-based therapeutics for transition to preclinical and clinical development.

## Challenges to Further Development of EV Therapies

**FIGURE 1:**
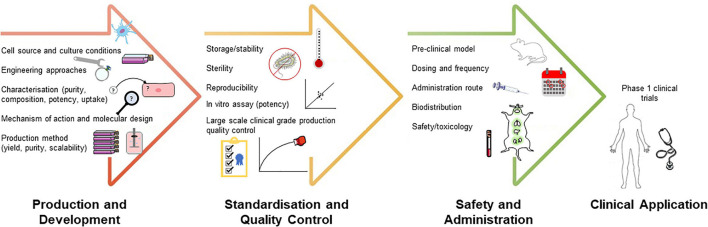
Challenges and translational considerations of development of EV-based therapeutics. Developing rational targeting or therapeutic strategies for these different stages will advance the application of EV-based therapeutics.

With increasing attention on EV-based therapeutics, the need for further refinement and standardization of design, production, and clinical administration approaches is critical. Specifically, there remain several fundamental challenges the field must come to terms with; low yield of production, scalable and standardized EV generation, standardized dose and potency monitoring, determination and quantification of molecular bioactivity for regulatory purposes, and unsatisfactory targeting capacity. Fortunately, international efforts to address aspects of these are ongoing (Lener et al., 2015; Reiner et al., 2017; Witwer et al., 2019; Gandham et al., 2020; Nguyen et al., 2020; Nieuwland et al., 2020; Rankin-Turner et al., 2021). In this section, we outline the challenges faced in EV therapeutic development, with an emphasis on the updated research and technologies offering avenues for preclinical and clinical advancement (Figure 1).

### Scalability and Standardization of EV Production

Clinical translation of EVs demands the development of standard, scalable, and cost-effective approaches for their production. For EV-based therapeutics, manufacturing requires high capacity and scalability without influencing the composition or potency of EVs (Whitford and Guterstam, 2019). A lack of clinical grade purification protocols suitable for large-scale production, and an incomplete understanding and standardization of variables influencing EV production represent main challenges in this area. Selection of an appropriate donor cell for native EVs (Charoenviriyakul et al., 2017) and monitoring variables such as growth state (epithelial, mesenchymal, or adherent/anchorage-dependent) (Tauro et al., 2013; Willms et al., 2016) can assist with this. Owing to significant functional advantages in regenerative medicine including wound healing and tissue repair, and therapeutic advantages in anti-inflammatory and low immunogenicity properties, amniotic and adipose cell-derived EVs are considered as a suitable candidates for therapeutic EV research and translation (Tan et al., 2018; Shukla et al., 2020). Aspects of culture influencing cell state include type of cell culture system [conventional and bioreactor systems (Mitchell et al., 2008; Mendt et al., 2018; Palviainen et al., 2019)] and media/supplements used (product and batch variance) (Quah and O’Neill, 2007; de Jong et al., 2012; Li et al., 2015; Patel D. B. et al., 2017; Thery et al., 2018; Zhu et al., 2021a). Moreover, modifications in culture parameters resultant of scaled-up systems, pH, mass transfer, and hydrodynamic (or shear) force, can result in modified cell state and growth, viability, expression, and activity of cell receptor/signaling, potentially impacting the composition and therapeutic efficacy of derived EVs. Further strategies exist to stimulate EV production and enhance yield have been reported, including *N*-methyldopamine and norepinephrine (Wang J. et al., 2020). However, the extent to which these factors impact EV composition, efficacy or other factors associated with their therapeutic use remains predominantly unknown (Colao et al., 2018; Adlerz et al., 2020) [reviewed in Whitford and Guterstam (2019)]. Therefore, as the therapeutic effects of EVs can be modulated by these variables, there is an emphasis on parental cell characteristics that should be carefully considered to exploit their clinical application (for example, therapeutic potency, immunogenicity, targeting selectivity), including their capacity to be manipulated (i.e., genetic engineering, transfection efficiency, genetic stability) or parental cell immortalization. For primary and immortalized cells, a thorough, risk-based analysis must be conducted, for the cells and their derived EVs, in addition to modified cells [reviewed in Rohde et al. (2019)]. In the context of EVs for therapeutic use, parental cell immortalization has been shown to enable sustainable production of EVs without influencing their therapeutic efficacy or immunosuppressive activity (Chen et al., 2011), however, safety concerns have been raised for the procedures and requirements to monitor oncogenicity and genetic drift [reviewed in Herrmann et al. (2021) and Xunian and Kalluri (2020)]. Recently, the production of therapeutic EVs has been amplified (Cha et al., 2018) by enhancing the biological functions of MSC-aggregates (spheroids) and their large-scale EV production (Cha et al., 2018). This study further highlighted key requirements in quality control (QC) monitoring cell source throughout changes in production and maintenance, monitoring morphological, size, and structural characteristics of derived EVs, their cytokine and miRNA expression and *in vitro* function, demonstrating they retained their stemness and marker gene expression during dynamic culture (Tan et al., 2018; Shukla et al., 2020). A safety concern with stem cells is the use of animal-derived serum for cell growth; the presence of such cross-species factors may cause issues from a regulatory standpoint in the production of therapeutics. Alternatively, using xeno-free culture media components or EV-depleted serum should be considered and influence on the compositions or physiological properties of derived EVs should be monitored. Accumulating evidence indicates that other naturally-derived EV source, including plant-based (NCT01668849, NCT01294072) (Dad et al., 2021) and bovine-milk-derived EVs (Grossen et al., 2021) may be sustainable alternatives for large-scale utility of engineered EVs. Bacterial EVs from non-pathogenic or probiotic bacterial source may also be harnessed as potential EV-based delivery carriers for anti-inflammatory function (Kuhn et al., 2020), with further advantages in their versatility in being readily functionalized (Shehata et al., 2019; Cheng et al., 2021) and their scalable production (Gujrati et al., 2014; Cheng et al., 2021).

For EV-based therapeutics to be considered a viable option for clinical applications, large-scale commercial production is required. For small-scale manufacturing, cells can be expanded in multilayered flasks, spinners, wave bags or fixed-bed or hollow-fiber bioreactors depending on their growth characteristics, while for large-scale culture, cells can be grown in large-capacity enclosed (stirred-tank) bioreactors or platform-rocker wave bags (Whitford and Guterstam, 2019). Presently, large-scale production of functional homogeneous stem cell-derived EVs for therapeutic utility has been developed using filtration (0.2 μm) and conventional ultracentrifugation (Mendt et al., 2018), and size-based chromatography fractionation (Reiner et al., 2017). While large-scale production of GMP-grade EVs for clinical trialing employs a combined approach of ultrafiltration coupled with sucrose/deuterium oxide (with ultracentrifugation cushion) (Lamparski et al., 2002; Nikfarjam et al., 2020). Further, GMP-grade EVs from human cardiac progenitor cells for therapeutic application have been isolated from 8 L of bioreactor-derived culture medium using tangential flow filtration (TFF) (without ultracentrifugation) in an integrated closed circuit which encompasses the full downstream process (clarification, concentration, diafiltration, and final sterilizing filtration) (Andriolo et al., 2018). This approach provides a high product yield (≥58%, 3 × 10^13^ particles), formulated in a clinical grade solution (Plasma-Lyte A) with concomitant consistent reduction of contaminants (total protein removal 97–98%). Importantly, this study employed a characterization QC strategy, including monitoring with suitable sensitivity, precision, and accuracy, according to regulatory guidelines. Commercially, companies including Codiak Biosciences employ TFF perfusion or alternating TFF perfusion to increase cell viability and isolate MSC-derived EVs using sequential filtration (0.8/0.45 μm), while others such as Evox Therapeutics Inc., use a combination of ultrafiltration and size-exclusion liquid chromatography. Larger-scale versions of other methods to isolate and enrich EVs (and subtypes within) may be possible with continuous-flow ultracentrifuges, size exclusion/ligand-activated core-bead technologies using distinct multimodal chemistry, microfluidics, field-flow fractionation, precipitation, continuous chromatography, and other industrial equipment, but feasibility of their use for EVs is limited (Li X. et al., 2019; Whitford and Guterstam, 2019; Yang et al., 2020; Martinez-Greene et al., 2021).

Profiling EV composition is a key strategy in understanding the influence had by modification and production variables. Technologies central to these efforts include mass spectrometry and nucleic acid sequencing. Indeed, proteomics can provide validation of modified (fusion) protein transfection in donor cells and act as a QC metric to evaluate the expression of donor cell and EV proteome and different EV/non-EV markers (Greening et al., 2017; Thery et al., 2018). Furthermore, proteomics can be employed with other biophysical/biochemical approaches to define and monitor the impact of culture media formulation from a single cell source on EV composition and surface protein epitope expression (Zhu et al., 2021a). Additionally, non-targeted metabolite profiling can assess how the metabolomic composition of EVs is influenced by conventional cell culture and bioreactor cell growth conditions (Palviainen et al., 2019). Proteomics and RNA sequencing has also been employed to indicate that cellular architecture (i.e., 2D vs. 3D) modifies transcriptomic and proteomic cargo of EVs (potentially altering overall function), the latter of which may affect efficiency of association and consequently uptake by recipient cells (Rocha et al., 2019). With significant therapeutic implications, the influence of engineered microtissues and immunoisolation devices on EV composition has recently been reported (Kompa et al., 2021; Millan et al., 2021), where microtissue stem cells cultured in a TheraCyte device 3D environment increased EV yield while additional markers (not detectable in EVs secreted by cells cultured in standard 2D conditions) were identified using proteomic profiling. Such findings reveal that cell growth conditions and media formulations influence the yield, surface epitopes (including tetraspanins), and broad (proteome/transcriptome/metabolome) composition of derived EVs. Thus, these are critical considerations in the scalable manufacture of clinical-grade EV therapeutics, as such variables may have profound impact on their utility. For instance, modifying the abundance of transmembrane proteins such as MHC complexes on their surface may affect their low immunogenicity (Petersen et al., 2011; Robbins and Morelli, 2014; Wahlund et al., 2017), a characteristic important for the utility of EVs as therapeutics.

As complex entities, EV therapeutics will require a suite of QC measures for successful clinical translation. A combination of discovery and pre-selected/targeted “omic” analyses will enhance our understanding of bioactive EV composition, cargo selection/loading; such knowledge could be translated to monitor generation, modification, and manufacture of EV-based therapeutic products with high throughput, sensitivity, and specificity. Indeed, in-depth monitoring is essential as EV preparations for preclinical application; often most studies use a combination of EVs (subtypes) in addition to various cell-derived and extracellular elements (i.e., secretomes) [reviewed in Rohde et al. (2019) and Witwer et al. (2019)]. For the successful translation of EVs to the clinic, the identification of critical quality attributes (e.g., size, purity, molecular composition) that impact the potency and stability of the product is essential (Figure 1). Considering the difficulties in standardized isolation and characterization of EVs, another strategy would be to prioritize therapeutic efficacy over purity (Wiklander et al., 2019; Herrmann et al., 2021). Recently, engineered recombinant EVs and reference particles have been suggested as reference materials to monitor technical variability of EV generation and their applications and promote intra- and inter-laboratory studies (Kim M. S. et al., 2018; Varga et al., 2018; Geeurickx et al., 2019, 2021). This could be applied to manufacturing and scalability in generating and monitoring EVs.

Increased and sustainable EV production is essential for the successful application and development of EV-based therapeutics. Recently, cellular nanoporation has been shown to increase production of exosomes as a universal nucleic-acid carrier for applications requiring transcriptional manipulation [up to 50-fold greater exosome yield and >103-fold increase in exosomal mRNA transcripts (Yang et al., 2020)]. Further, M-NVs generated by microfluidic and extrusion techniques have a high and consistent production yield, in comparison to native EVs, overcoming their incumbent production and isolation challenges and represent a promising alternative to native EVs for scalable and clinical application. Considerations of such approaches in terms of physical parameters may present new difficulties in establishing a standardized protocol for their generation, efficiency of molecular incorporation, modification, and storage (Shah et al., 2019; Meng et al., 2021). Further, M-NVs are comprised of diverse, undefined molecules derived from donor cells, which may cause potential safety concerns, similar to native EVs. As such, a careful screening of producer cells is essential—focused on the consistency of long-term cell growth, and specific therapeutic potency and bioactive effect. In addition, the ability of M-NVs post-generation to encapsulate drugs (i.e., hydrophilic, negatively charged macromolecular drugs) remains inefficient (generally < 30% incorporation), highlighting the need to further refine composition to enhance encapsulation efficiency (Yoon et al., 2015; Molinaro et al., 2016).

Key criteria in the development of EV-based therapeutics is the stability, preservation and storage of EV therapies (Jeyaram and Jay, 2017; Kusuma et al., 2018). Recently, the effects of storage conditions on EVs for functional analysis and therapeutic use was investigated (Wu et al., 2021), evaluating temporal EV stability/quantity across various storage temperatures and freeze-thawing cycles. Importantly, storage alters EV size distribution and impacts cellular uptake and biodistribution, with −20°C (short-term) and −80°C (long-term) storage recommended. Further, for GMP-compliant EVs, several studies have reported native EV storage at −80°C in single-dose aliquots (preliminary stability studies indicate no loss of functional activity in up to 7 months) (Andriolo et al., 2018) and modified EVs storage at –80°C for up to 5 months with no change in potency or activity (Mendt et al., 2018).

Overall, challenges of industrial scale-up and specific variables that will impact transition to clinical development must be addressed and monitored in early development. The clinical and commercial demands of EV-based therapeutic production such as high, consistent yield, reproducible composition/purity, and efficiency can eventually be met with modifications to existing technologies for improved scalability (Colao et al., 2018; Adlerz et al., 2020).

### Analytical Assessment of EVs: Potency and Molecular Insights for Regulatory Purpose

In delivering EV-based therapeutics dosing remains a significant challenge, with dose selection, assessment, and administration (route, frequency, time window) all factors in achieving therapeutic benefit without adverse effects. Development of appropriate potency assay that employ relevant functional end points (Nguyen et al., 2020), and demarcate *in vitro* potency (dependent on EV property and cell type) and *in vivo* efficacy is required (Willis et al., 2017; Witwer et al., 2019). Such potency assays need to be standardized and context-/disease-/model-dependent, which are unfortunately currently limited in their acceptance and utilitization (Adlerz et al., 2020).

Analytical assessment and monitoring, and rigorous *in vitro* and *in vivo* testing for safety and efficacy must precede approval of EV-based therapeutics [as comprehensively discussed (Reiner et al., 2017; Nguyen et al., 2020)]. Several seminal clinical-grade EV studies have reported specific potency assessments including *in vitro* anti-/pro-activity, sterility, *in vivo* pro- activity, and toxicity or immunogenicity assays (Andriolo et al., 2018; Mendt et al., 2018). Analytical assessments include: (i) molecular fingerprinting (identify bioactive/target molecule/s), monitoring of multiple components and influence of preparation/isolation method on donor and cell-derived product), (ii) potency assays (Nguyen et al., 2020) (monitoring therapeutic effect *in vitro*/*in vivo*), and (iii) mechanistic assays (identify mode of action) (Willis et al., 2017; Colao et al., 2018; Surman et al., 2019; Witwer et al., 2019). Such analytical assessments may provide quality control in the transition from research-grade to clinical (GMP)-grade EVs [reviewed in Adlerz et al. (2020) and Willis et al. (2017)].

At present there are no standardized methods in quantifying EV concentration and dosage. Moreover, comparative studies of EV monitoring techniques has shown that protein concentration is not relative to particle number (Lobb et al., 2015); consequently such criteria is insufficient to determine dosing. Quantitative EV analytical methods include reporting cell equivalents, protein concentration, and quantitative analytical measurement [dynamic light scattering (DLS), single-particle interferometric reflectance imaging sensing, tuneable resistive pulse sensing (RPS), nanoflow cytometry and nanoparticle tracking analysis (NTA)] (Arab et al., 2021). Significant concerns have recently been presented around existing single-particle analysis capabilities (e.g., sizing, counting, tetraspanin phenotyping) (Arab et al., 2021). Moreover, single-particle technologies will be required to separate heterogeneous EV populations into well-defined and easily recognized subgroups. Therefore, consideration of limitations in some analytical platforms is a key requirement [e.g., resolution and accuracy of the quantitative analysis of EVs using single-particle analysis capabilities (Welsh et al., 2020; Arab et al., 2021)]. In order to fully characterize a therapeutic nanoparticle preparation, it is imperative to consider particle size, particle size distribution, charge, number of particles or concentration, and the molecular composition (Nelson et al., 2020). Absolute values measured for particle or protein concentration thus need to be critically evaluated and compared to control condition, using calibration/standardized measurements at specific dose as required. Current methods have shown to be efficient in quantifying the biophysical parameters [light-scattering methods (multiangle light scattering, DLS, NTA, particle interferometric reflectance imaging sensing), RPS, transmission- and cryogenic-electron microscopy, and small angle neutron scattering], hence providing the tools to validate and standardize nanoparticle therapeutics (Buzas et al., 2017). It should be noted, these technologies are not able to determine the particle type or chemical/molecular makeup of a sample, making it difficult to determine whether the sample contains EVs, protein aggregates, or other non-membranous particles (Arab et al., 2021). This limitation requires optimization of sample purity prior to size measurement.

Expectantly, EV heterogeneity confounds aspects of therapeutic application, with subtypes varying in composition and function. One of the challenges of EV biology is identification of specific EV-subtype markers (Thery et al., 2018). If aiming to purify EVs for stringent biochemical analysis (e.g., define cargo for regulatory purposes, quality control, therapeutic design) and specific functionality (e.g., identification of bioactive cargo, therapeutic screening), then rigorous purification and fractionation strategies are critical [reviewed in Gandham et al. (2020); Xu et al. (2016)]. Current research indicates that further subdivisions of EVs are needed to accurately differentiate subtypes (e.g., biogenesis, size, charge, molecular cargo), which may in turn offer unique therapeutic avenues. A key advantage here is the considerations of quality assessment and quantification of EV preparations based on recommendations of MISEV2018 and EVTRACK (Consortium et al., 2017; Thery et al., 2018). These considerations will be essential for generation of clinical-grade EVs or standardized engineered EVs (Lener et al., 2015; Merino-Gonzalez et al., 2016; Paganini et al., 2019; Meng et al., 2020; Salmond and Williams, 2021) (Figure 1).

To establish consistency in the therapeutic assessment of clinically translated EVs, effective dosing harmonization is a key requirement. It is recommended to consider the type of payload, whether it is a drug (Doxorubicin, Aspirin, Gemcitabine), an enzyme/protein (caspase, TTP1) or other biological component (nucleic acids, lipids). It is known these groups possess different pharmacokinetics and thus different units have been assigned to describe their activity or effect i.e., drug dosing is expressed in terms of mass of active substance (μg, mg, g) per pharmaceutical presentation unit (capsule, ampule, particles, grams) (Powell et al., 2021); enzymes and protein dosing is expressed in terms of enzymatic catalytic activity or IU (1 IU = 1 μmol/min = 1/60 μmol/s ≈16.67 nmol/s) (Units of Enzyme Activity, 1979; Iupac, 2018); lastly, biological payload such as nucleic acids and lipids could potentially be expressed in units of mass or activity depending on their function. Another consideration is the function and effect of the native/engineered EVs itself, aside from specified cargo. As vesicles interact with the host, proper controls should be established to determine their protective [i.e., RNAse-mediated degradation effect (Mendt et al., 2018)] and measured effect without the cargo of interest (Kennedy et al., 2020). Studies have shown that dosing variations of purified EV (from 30 to 300 μg of protein) did not present any adverse consequences and still exerted their desired effect in a myocardial infarction model *in vivo* (Barile et al., 2014). This suggests the key for dosing is not the particle itself but the cargo, especially if incorporated post-isolation.

As EVs are biological entities, their complex and variable nature challenges the highly regulated requirements for pharmaceutical production. A solution to this is functional assessment of each batch [cell source consistency (Viaud et al., 2011; Andriolo et al., 2018; Mendt et al., 2018) and EV production for specific patient-derived cell source based on EV yield (Mendt et al., 2018), positive/negative EV markers (Andriolo et al., 2018; Mendt et al., 2018), reviewed in Nguyen et al. (2020)]; identification and quantification of key markers indicating functional competency could aid this process (van Balkom et al., 2019) [i.e., tissue-based immunoregulation (Ma et al., 2020)]. Mapping specifically regulated proteins using microfluidic approaches (Zhao et al., 2016; Fang et al., 2017; Xu et al., 2018) or quantitative proteomics onto known protein networks has highlighted mechanism of action (Martin-Jaular et al., 2021) and is being applied to EV research (manufacture, composition, and function). Proteomic approaches allow researchers to understand protein signatures of native and engineered EVs (Nasiri Kenari et al., 2019; van Balkom et al., 2019), which may have implications in quality control platforms to confirm the identity and test for purity of therapeutic EVs. Proteomics has been used to assess plasma EVs after separation from lipoproteins (i.e., lipoprotein particle depletion) (Karimi et al., 2018), tissue-derived stress/damage markers following cardiac-EV isolation (Claridge et al., 2021), and oncogenic mutations on EV proteome landscape (Al-Nedawi et al., 2008; Lobb et al., 2017; Emmanouilidi et al., 2019; Chennakrishnaiah et al., 2020; Shafiq et al., 2021; Tawil et al., 2021). Several key omic-based studies have provided direct insight into the composition and (re)classification of EVs, their biogenesis and content (Greening et al., 2015; Kowal et al., 2016; Jeppesen et al., 2019; Kugeratski et al., 2021; Martinez-Greene et al., 2021; Rai et al., 2021). Moreover, an aptamer-based proteomic analysis (SOMAscan) has enabled multiplexed, highly-sensitive, and specific protein detection in human blood and other biomatrices, and applied to profile EVs (Welton et al., 2017). Other high-throughput MS approaches, such as multiple/parallel reaction monitoring MS (MRM/PRM-MS) enable rapid and accurate identification and quantification of protein biomarkers in broad dynamic range (Anwar et al., 2019; Makridakis et al., 2020). Indeed, MRM-MS has been used for identification of *Mycobacterium tuberculosis* peptides in patient derived exosomes (Kruh-Garcia et al., 2014), highlighting the sensitivity and specificity of this approach in EVs. Additionally, RNA profiling has been used to confirm the absence of inhibitory miRNAs (Rohde et al., 2019).

The application of multi-omic technologies, such as transcriptomics and proteomics are emerging as crucial for understanding how EVs perform therapeutic functions (Li et al., 2021d). In the wider drug development field, omics technologies are already in use to better understand mechanisms of action and identify off-target effects (Jia et al., 2019; Friman et al., 2021). Additionally, comprehensive profiling of patient and population proteomes/transcriptomes contribute to *in silico* predictions of drug efficacy [reviewed in El-Khateeb et al. (2019)]. Integration of cellular technologies [microphysiological systems or “tissue chips” (Ronaldson-Bouchard and Vunjak-Novakovic, 2018; Low et al., 2021; Zhang S. et al., 2021)] aimed to closely mimic *in vivo* conditions to assess the potency of experimental biological products and understanding of tissue-specific effect will be important future developments in clinical translation. The development of new technologies, such as machine learning and control algorithms, to interface with microphysiological systems may further advance their use in systems biology and precision medicine (Zhang Y. S. et al., 2017). For clinical translation, the FDA does not require any understanding of the mechanism by which a drug acts, moving directly to clinical trials without this knowledge may be a clear path. However, often limitations in understanding how the drug or biological regulator works may contribute to unfortunate outcomes, off-target/side effects, or poor/ineffective dosing; understanding mechanism of action may help to stratify/focus clinical trials to those patients most likely to respond (No Author, 2010). Significant changes in the FDA (Critical Path Initiative) now support development of patient screens to improve the chances for drug approval, and integration of pharmacogenomic data (proteomics, metabolomics, genome-wide association study data) in developing and evaluating medicines. In the context of EV-based therapeutics, identification of active components as a constituent of the total EV will enable optimization and improvements in dosing and effect (Xin et al., 2017; Jia et al., 2021).

### Tissue Targeting and Delivery of Therapeutic EVs

Specific, targeted delivery presents a major challenge for EV-based therapeutics. Sophisticated solutions are required to minimize off-target accumulation and maximize efficacy through the targeted transport and delivery of cargo (Vargason et al., 2021). Indeed, a major obstacle preventing widespread usage of other types of regulatory therapeutics (i.e., nucleic acid polymers/oligonucleotides) is efficient delivery to target organs and tissues, minimizing off-target accumulation [reviewed in Roberts et al. (2020)]. In nanoparticle-based therapeutics, the pharmacokinetics, biodistribution and targeting are all influenced by design and biological factors of the nanoparticle constructs (Haun and Hammer, 2008; Calderon et al., 2009; Haun et al., 2011; Gu W. et al., 2019) [reviewed in Mitchell et al. (2021) and Wei et al. (2018)]. Additionally, different cell types and phenotypes internalize and traffic nano-carriers differently (MacParland et al., 2017; Patel S. et al., 2017; Brownlee and Seib, 2018) [reviewed in Biswas and Torchilin (2014) and Howard et al. (2014)]. This is especially relevant for EV therapeutics, as the heterogeneity of EVs can contribute to differential recognition and binding specificity and target cell uptake (Chivet et al., 2014; Choi et al., 2019) [reviewed in Mathieu et al. (2019)]. Characterization of such mechanisms will be essential in the field’s pursuit of accurate, potent, on-target delivery and uptake of EV therapeutics.

Drug targeted delivery can be passive, where circulating nanoparticles [e.g., coated with platelet or red blood cell membranes (Usman et al., 2018)], extravasate through leaky vasculature and accumulate in surrounding tissue *via* the enhanced permeability and retention (EPR) effect (Chan et al., 2010; Hu et al., 2011; Howard et al., 2014; Luk et al., 2016). Alternatively, targeting can be active, relying on membrane moieties to bind protein/peptide receptors/sequences on target cells or tissues (Peer et al., 2007; Bertram et al., 2009; Cheng et al., 2020; Yang et al., 2020). The relatively large surface area to volume ratio of EVs enables highly efficient surface interactions, highlighting the latter form of targeting as a promising avenue for development. Recently, the inflammation-targeting ability of platelet-derived EVs (PEVs) was verified, with drug-incorporated PEVs able to target inflamed lungs *in vivo* to precisely deliver and modify the immune response at the site of inflammation (Ma et al., 2020), revolutionizing the way inflammatory diseases can be treated. While effective, the molecular interactions (perhaps surface-based) behind this functional delivery are not yet understood. However, there are known protein signatures on EV surfaces which direct uptake in specific organs (Hoshino et al., 2015); unfortunately, very few targeting moieties have been identified and characterized on EVs. Defining additional signatures is essential for the refinement of targeted EV therapeutics, and will require analysis of their surfaceome and biodistribution, as well as the molecular signatures of proteins/peptides exposed in the targeted tissue/cell pathology [reviewed in Howard et al. (2014) and Shao et al. (2018)].

The surface proteome of EV subtypes (exosomes) has been defined using various proteolytic and biotinylation approaches (Cvjetkovic et al., 2016; Williams C. et al., 2019; Xu R. et al., 2019) with their topology determined using similar methods (Jeppesen et al., 2019). This has provided insights into components exposed and available for cell-interaction. Recently, proteins with exposed regions on the EV surface, CD63 and LAMP2B, have been the base for topologically distinct scaffolds for fusion proteins containing targeting sequences, enabling the flexible engineering of EV surface for applications in disease-targeted drug delivery and therapy (Curley et al., 2020). Use of these fusion protein scaffolds of attachment of components with known binding partners enriched at target sites has successfully assisted in increased targeted uptake, but has not abolished non-specific uptake (Munagala et al., 2021). Development of new scaffolds is dependent on the display of a N- or C-terminal on the outer surface of EVs, with topology of the surface proteins essential (Jeppesen et al., 2019). However, the surface signature of EVs is highly heterogeneous, which presents a challenge in designing targeting scaffolds present on all EVs, rather than a subtype, and could reflect different baseline functionalities (Kowal et al., 2016; Xu R. et al., 2019). Furthermore, despite the high lipid content and diversity on EVs, the role of lipids (i.e., glycosphingolipids) in drug delivery, specific tissue-derived EVs (Flaherty et al., 2019), and how other surface moieties facilitate delivery (Zhu et al., 2018; Mentkowski and Lang, 2019) is still poorly understood. Comprehensive multi-omic surfaceome studies of homogeneous EVs will assist in understanding which effectors (e.g., tetraspanins, integrin receptors/ligands, glycoproteins, membrane lipids) facilitate direct functional content transfer and targeting, findings which will be critically important in exploiting EVs as drug delivery systems (Kooijmans et al., 2021; Richter et al., 2021). Combining surfaceome characterization with techniques to study tropism (Hoshino et al., 2015; Wu et al., 2020) and biodistribution (Wu et al., 2020) will assist in identification of components that determine targeting and interaction.

Understanding the biodistribution of EVs is critical for the identification of targeting-moieties and ensuring dosage at the intended site/tissue. While simple, fast, and relatively inexpensive to use, lipophilic dyes can present issues for biodistribution studies. Dyes can form micelles (Puzar Dominkus et al., 2018) and interfere with EV size, charge, uptake, biodistribution and clearance (Lai et al., 2014; Dehghani et al., 2020), raising questions about their suitability for this application (Takov et al., 2017). Indeed, EVs are likely degraded and/or recycled *in vivo* resulting in inaccurate spatiotemporal information (Takov et al., 2017)—a consideration when using such dyes is their capacity to interact with other membranes and remain visible in such tissues. Fortunately, alternatives to dye-based tracking are emerging; recent developments in live cell reporters allow visualization of EV biogenesis, uptake and intracellular trafficking (Sung et al., 2020). Fusing surface proteins to NanoLuc or ThermoLuc allow highly sensitive *in vivo* quantification or real time imaging, respectively, at low cost and in semi-high throughput (Kojima et al., 2018; Gupta et al., 2020). Additionally, other reporter systems have been used to label EVs for *in vivo* imaging, spatiotemporal dynamics, and pharmacokinetic analysis of administered EVs (Takahashi et al., 2013; Lai et al., 2014; Sung et al., 2020; Wu et al., 2020). Imaging technologies have shown EV biodistribution is influenced by route of administration (intravenous, intraperitoneal, and subcutaneous injection) and dosage (Ohno et al., 2013; Takahashi et al., 2013; Wiklander et al., 2015). Mode of delivery also effects the plasma pharmacokinetic patterns of EVs, while different subpopulations of EVs differ in their *in vivo* biodistributions (Gupta et al., 2020). This variation presents a challenge for the clinical application of systemically administered EV therapies, with altered biodistribution complicating potential dosage and off-target effects. However, non-systemic administration of EVs may bypass this challenge.

Delivery of EV-based therapeutics directly to the site of interest overcomes the limitations of current therapeutic delivery strategies and can ensure sequestration of the therapeutic. Lung-based pathologies are employing inhalation as the method of administration to ensure site-specific delivery (NCT04389385, NCT04747574, and NCT04276987). Injections of EVs to sites of damage are another method of focusing delivery, with therapeutic application of EVs directly to arthritic joints (Wang et al., 2017; Wu et al., 2019; Li et al., 2021b), venous ulcers (NCT04652531), inner ears (Gyorgy et al., 2017), eyes (NCT03437759), and heart [damaged myocardium (Yao J. et al., 2021)]. While suitable for individual treatments at injection-accessible sites, ongoing release of EV-based therapeutics at inaccessible sites raises a challenge. The injection or topical application of hydrogels and biogels offer a form of slow-release EV administration directly to the site of injury (Li L. et al., 2020; Shi et al., 2021; Zhao et al., 2021; Zhu et al., 2021b). Furthermore, the recent development of EV-coated scaffolds, such as cardiac stents and cardiac fibrin patches, allow for the therapeutics extended release at the site of damage (Hu S. et al., 2021; Yao J. et al., 2021). As a minimally-invasive direct therapeutic delivery strategy, EVs have been shown to be released in high capacity *in vivo* from human stem cells within a semi-permeable chamber inserted subcutaneously for cardiac therapy (Kompa et al., 2021). Coating implants for expedited healing and recovery is an area with great therapeutic potential, however, refinements and standardization will be required prior to clinical translation. Developments utilizing shape memory materials [e.g., smart or intelligent materials (Huang et al., 2019; Zhao et al., 2019)] and dynamic response composites which can adapt to external stimuli, could offer significant advantages in EV-coated implant/scaffold systems for wound treatment (Melocchi et al., 2021). Further improvements to EV-based therapeutic delivery will be made with additional molecular characterization of EV-tissue tropism, biodistribution, and delivery mechanism designs to ensure the specificity of therapeutic delivery for specific cell types.

## Conclusion

Extracellular vesicle-based therapeutics hold the greatest clinical promise when a combination of native and engineered aspects are utilized. Native EVs hold innate therapeutic potential—they are biocompatible, stable, and due to their specific targeting, facilitate therapeutic use. However, there are significant challenges associated with their commercialization and clinical development, with engineered EVs allowing modified content, increased production, and targeting for improved therapeutic outcome. Regardless of native or engineered state, there are several aspects which must be considered prior to pharmaceutical translation and clinical application (Figure 1), primarily the source, standardized EV generation/isolation and characterization, defining composition/potency/dose/safety, understanding targeting and biodistribution, and elucidating mechanism of action in target cell(s). This review details diverse scalable processes/strategies for EV generation and isolation, and development/integration of methods for research/clinical grade quality control. In developing EV-based therapeutics, a quantitative analysis of pharmacokinetics, biodistribution, and influence of storage conditions on shelf life, are essential. Indeed, several seminal studies have reported a clinically feasible approach for EV production, scalability, and storage for therapeutic application (Gimona et al., 2017; Andriolo et al., 2018; Mendt et al., 2018; Witwer et al., 2019). Interestingly, for EV-based therapeutic development, as discussed (Grangier et al., 2021), the most advanced scale-up strategies preferentially use abundant quantities of available biomaterials (biofluids, blood products including plasma, red blood cells, platelets) and produce engineered EVs or secretome-based products for translation. Such strategies may be the result of the clinical translation of prior regulatory requirements in the established, licensed use of these products as medicines, abundant supply, human origin, and biotechnology and cell therapy industry links to accelerate clinical translation. The use of these products has significantly inherent translational advantages for hemostasis, for regenerative medicine, and as drug-delivery vehicles (Johnson et al., 2021). Once quality control concerns have been addressed and applied by the field, clinical trials will be able to advance further (Gandham et al., 2020). Successful translation of EV therapeutics in immunomodulation, regeneration and repair, and combination therapies will provide desperately needed treatments globally, transforming the current bio-pharmaceutical landscape.

Considering that EVs carry and transfer various functional molecular cargo, quantitative global analyses to understand EV-associated components [e.g., EV-mediated transfer of proteins/lipids/RNAs between specific cell types and organs (Costa-Silva et al., 2015; Hoshino et al., 2015; Flaherty et al., 2019; Rodrigues et al., 2019; Kugeratski et al., 2021; Nguyen et al., 2021)] warrants further investigation. Increasingly, the field is shifting toward systems biology to understand EVs (Xu et al., 2016; Gezsi et al., 2019), integrating different analysis platforms to achieve multi-omic characterization of EVs for therapeutic application—their source (different donor origins, organ/tissue-derived), composition (including core and surfaceome/interactome landscape), and capacity to reprogram target cells and phenotype (Hoshino et al., 2015, 2020; Melo et al., 2015; Kowal et al., 2016; Xu et al., 2016; Figueroa et al., 2017; Greening et al., 2017; Flaherty et al., 2019; Rontogianni et al., 2019; Xu H. et al., 2019; Jung et al., 2020; Bijnsdorp et al., 2021; Kugeratski et al., 2021; Rai et al., 2021). Further development of these technologies will facilitate the advancement and refinement of EV-based therapeutics. Indeed, developments in EV-based function in cross-kingdom delivery [i.e., RNAs in trafficking from plant to regulate fungal pathogens (Cai et al., 2018)] may further develop effective delivery methods of pathogen-targeting regulatory RNAs for therapeutic and agriculture use.

Designing EV-based therapeutics is reliant on identification of components with a beneficial effect at a target site. Given their complex composition, mechanisms by which EVs induce their therapeutic effects remain incompletely understood. As a priority, a major challenge toward therapeutic utility of EVs is their heterogeneity in content and composition inherent in their biogenesis and generation. This heterogeneity currently complicates the design, dose, standardization, regulation, and delivery of EV-based therapeutics. Enrichment strategies that can distinguish between different EVs may help identify the functional sub-population and enrich for active components. Such an approach will not only result in more potent therapeutic applications but allow decoding and translation of molecular insights and mechanisms of action underlying function. The design of simple, effective, and cost-efficient processes to assess the required purity of EVs will facilitate much-needed standardization in the field. Careful consideration and standardized/regulatory requirements for challenges raised in this and other key reviews (Willis et al., 2018; Whitford and Guterstam, 2019; Wiklander et al., 2019; Gandham et al., 2020; Nguyen et al., 2020; Rankin-Turner et al., 2021) will assist in development of EV-based therapeutics, bringing them closer to the clinic (Davis et al., 2008; Chan et al., 2010; Buss and Bhatia, 2020; Li et al., 2021c; Swingle et al., 2021; Yang et al., 2021).

## Author Contributions

BC, JL, and QP contributed equally to all aspects of the design, writing, figure generation, and editing of this manuscript. DG contributed to the design, writing, and editing of the manuscript. All authors approved the submitted version.

## Conflict of Interest

The authors declare that the research was conducted in the absence of any commercial or financial relationships that could be construed as a potential conflict of interest.

## Publisher’s Note

All claims expressed in this article are solely those of the authors and do not necessarily represent those of their affiliated organizations, or those of the publisher, the editors and the reviewers. Any product that may be evaluated in this article, or claim that may be made by its manufacturer, is not guaranteed or endorsed by the publisher.
